# Towards a Trait-Based Approach to Potentiate Yield under Drought in Legume-Rich Annual Forage Mixtures

**DOI:** 10.3390/plants10091763

**Published:** 2021-08-25

**Authors:** Susana T. Leitão, Mara Lisa Alves, Priscila Pereira, Aziza Zerrouk, Bruno Godinho, Ana Barradas, Maria Carlota Vaz Patto

**Affiliations:** 1 Instituto de Tecnologia Química e Biológica António Xavier, Universidade Nova de Lisboa (ITQB NOVA), Avenida da República, 2780-157 Oeiras, Portugal; mara.l.alves@gmail.com (M.L.A.); priscilaaful@gmail.com (P.P.); aziza.zerrouk@gmail.com (A.Z.); cpatto@itqb.unl.pt (M.C.V.P.); 2 Fertiprado, Herdade dos Esquerdos, 7450-250 Vaiamonte, Portugal; bgodinho@fertiprado.com (B.G.); abarradas@fertiprado.com (A.B.)

**Keywords:** grass–legume mixtures, climate change, resilient annual forage species, above and below-ground phenotyping, photosynthesis

## Abstract

Mediterranean annual forage mixtures are facing the impact of climate change, especially higher frequencies of winter-time drought. Increased mixture plasticity to climate variability is needed to mitigate this impact. However, little information exists regarding the specificities and complementarities of each forage species component to potentiate mixture resilience under drought. In this study, we identified traits with breeding potential under water scarcity through a detailed characterization of leaf and root-related parameters of 10 legume and grass species components of Mediterranean annual forage mixtures, complemented by their photosynthetic response evaluation under well-watered and water deficit conditions. This integrated approach also allowed us to identify the most resilient species to water deficit. In particular, we found that the highest canopy height and root to shoot ratio of grass components complemented well the highest aerial and root biomass and superior photosynthetic performance of the legume components. *Trifolium squarrosum* and *Triticosecale* showed the most adequate combination of traits and the best photosynthetic performance under water deficit within each species family. Although some of these traits are not commonly used in annual forage selection, they may in part explain the potential higher resilience of the grass–legume mixture under water deficit and should be considered in forage breeding.

## 1. Introduction

Grass–legume mixtures are commonly cultivated under dry Mediterranean conditions to increase forage productivity [1] while reducing the need for nitrogen fertilization and decreasing production costs and the carbon footprint of the agricultural system. Legumes are considered “greenhouse gas neutral” since all the carbon needed for their symbiotic nitrogen fixation comes directly from the atmosphere via photosynthesis [2]. The cereal components of the mixtures usually improve their yields and forage dry matter at the expense of this nitrogen increase in the soil. Additionally, grass–legume mixtures provide higher-quality animal feed than sole crops. Legumes are generally considered high-quality forage due to their low fiber and high protein content [3], with their relatively low energy concentration easily being compensated for with the cereal components of the mixture [2].

As predicted by climate change models [4], the whole Mediterranean region (including Portugal) will be seriously affected in the near future by significant changes in climate, such as warmer summers and a 40% loss of winter precipitation. On the other hand, flash floods, caused by short and locally heavy rains that sporadically affect this region, will be aggravated and may become more frequent [5]. The impact of climate change on the productivity of forage mixtures will depend on their resilience to this higher inter-annual climatic variability, in particular to drought, which in turn is closely related to the mixture’s biodiversity [6]. Not only the number of species, but also which species, their functional traits, and their interactions, shape the biodiversity of a grass–legume mixture. In this way, an optimal combination of plant species and traits conferring relevant adaptive strategies in response to water scarcity should be defined and explored according to the targeted environments.

In Portugal, the semi-natural annual forage mixtures are adapted to avoid dry hot summers by adjusting their life cycle to the seasonal water availability (winter rains). However, since this strategy could result in low yield [7], farmers are now much more attracted to growing optimized biodiverse annual forage mixtures rich in legumes. The use of these mixtures allows for the improvement of productivity, soil fertility, erosion control, and water retention [8]. This last aspect is due to an asynchronous species development of grass–legume mixtures that also leads to a better exploitation of available water in the soil in addition to a more permanent soil coverage. The complementary species root architecture contributes indirectly to a higher resilience of the mixture to drought throughout the growth cycle when compared to sole crops [9,10]. However, it is also clear that the drought tolerance levels of each species component by themselves may also contribute to increased resilience of the mixture. Thus, although each mixture’s components might respond differently when combined in different ways, it is important to understand the individual species performance under water deficit.

At individual plant level and under moderate drought, the maintenance of biomass production can be achieved primarily by maximizing soil water capture, while maintaining stomatal gas exchange and transpiration [11]. Thus, a deep root system with a high density of roots and the maintenance of leaf area, leaf relative water content, and photosynthetic capacity are traits associated with high yield under water-limited conditions [12]. In fact, root development and distribution affect plant growth and partially compensates for plants’ competition for light and carbon dioxide, leading to higher photosynthetic rates and crop yields [13]. Despite being more laborious to measure, root traits might be better predictors of soil and ecosystem processes than leaf traits [14]. Other individual plant strategies include high water use efficiency and low stomatal conductance through dense leaves and the investment in high root to shoot ratios [14].

Until recently, drought tolerance has not been actively targeted in annual forage mixture improvement, but due to climate change leading to increased water limitations, this needs to change. Breeding for mixtures that can maintain water and nitrogen uptake under water scarcity should be a primary objective for grassland and forage research [15]. However, to choose and optimize resilient mixture combinations, more knowledge on the mixture’s individual component performance under water-deficit conditions is required as a first step. There is also a need to understand which traits contribute the most to discriminate species performance and how the typical species components of these biodiverse mixtures rank or group for these water-deficit resilience traits.

In this study, we explored the complementarity/diversity of above and especially below ground phenotype and photosynthesis-related traits among annual forage species. For this, we performed a detailed individual characterization, not usual in forage breeding, of 10 grass and legume species that are typical components of Mediterranean biodiverse annual forage mixtures. Additionally, we compared the photosynthetic performance of the same 10 species under well-watered versus water deficit.

The present work will enable the identification of traits with breeding potential under water scarcity and of the most resilient individual species components as first steps to support the optimization of annual forage mixtures to cope with climatic constraints that are becoming increasingly frequent in the Mediterranean region.

## 2. Results

The present study was carried out to identify the most resilient species components of Mediterranean annual forage mixtures to water deficit as well as to highlight the resilient traits with higher breeding potential for these environmental conditions. For that, a detailed species morphological development characterization (9 to 10 weeks after germination) using aboveground leaf related (such as aerial biomass, plant length or specific leaf area) and below ground morphological traits (such as root and secondary root lengths and root fraction dry weight) was performed individually on 10 grass and legume species that are typical components of Mediterranean biodiverse annual forage mixtures. Additionally, the photosynthetic performance of the same 10 species under well-watered and water-deficit conditions was compared after seven weeks of initial development, assessing traits such as leaf relative water content, chlorophylls and carotenoids content, chlorophyll a fluorescence, and gas-exchange parameters.

### 2.1. Morphological Development Characterization

The grass species *Avena strigosa*, *Lolium multiflorum* 2n, *L. multiflorum* 4n, *Triticosecale* (Poaceae), and the legume species *Trifolium incarnatum*, *T. michelianum*, *T. squarrosum*, *T. suaveolens*, *T. vesiculosum*, and *Vicia villosa* (Fabaceae) were characterized using leaf and root-related parameters during the first weeks of development.

Significant differences were detected among the 10 plant species for all traits, except for tillering, at all analyzed time points (Supplementary Table S1).

When comparing the traits’ mean values among species for each time point (Supplementary Table S2), *V. villosa* stood out as the species with higher plant length as early as the third week after germination (WAG). In the opposite situation, the other legume species analyzed (*Trifolium* spp.) had the smallest plant length values. However, regarding canopy height, all the grass species (*A. strigosa*, *L. multiflorum* 2n and 4n, and *Triticosecale*) had higher values than the legume species between the 3rd and the 9th WAG.

*Vicia villosa* was also the species that invested the most in its aerial biomass dry weight (ADW) in the early stages of development. However, at 10 WAG, *T. michelianum* surpassed *V. villosa* ADW. A similar progression pattern was also observed for root dry weight (RDW). Considering the dry root to dry shoot ratio (R/S), the differentiation between the grasses and the legume species was clear. While the legume species always had a R/S ratio below 1, giving priority to shoot development, the grasses’ tendency was to allocate most of their biomass to the root. At the final time point (9 WAG), *A. strigosa*, *L. multiflorum* 2n and 4n had R/S values between 2.002 and 2.495 and *Triticosecale* was 1.891, whereas the values of the legume species ranged from 0.351 (*V. villosa*) to 0.809 (*T. squarrosum*) (Supplementary Table S2).

Regarding branching (B), a parameter measured only in legume species, *V. villosa* already at 3 WAG, and *T. michelianum* at 6 WAG, were clearly the two species with the highest levels of branching. *V. villosa* was also the species with the highest number of nodules (Supplementary Table S2).

**Figure 1:**
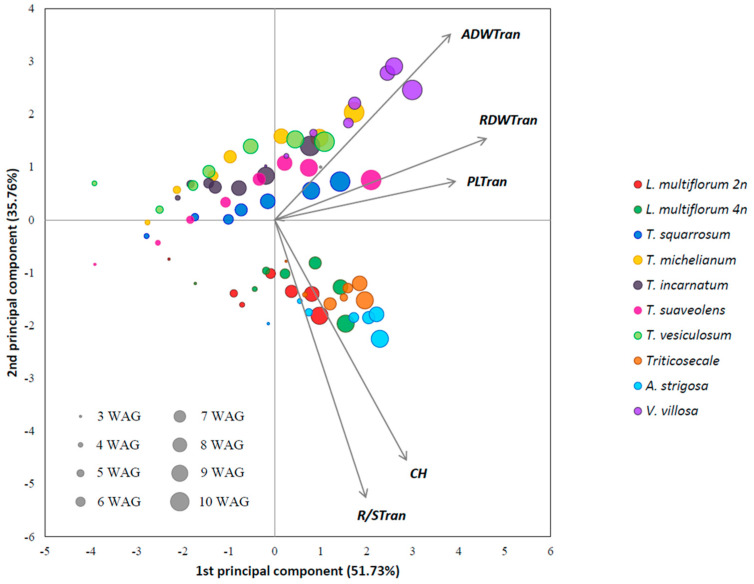
Principal component analysis of morphological data measured across time in 10 different annual forage species (grasses and legumes). The size of the circles indicates the time of measurement (weeks after germination, WAG). The two principal components accounted for 87.59% of the total variation. Traits’ abbreviations: ADW—aerial biomass dry weight, CH—canopy height, PL—plant length, RDW—root biomass dry weight, R/S—dry root to dry shoot ratio. The suffix “Tran” next to the traits’ abbreviation means that the trait values were transformed.

Figure 1 shows the clear separation between the grass and legume species across time by canopy height and R/S ratio. It was possible to discriminate among legume species by plant length and aerial and root dry weights, with *V. villosa* revealing a growth development distinct from the *Trifolium* spp.

**Figure 2:**
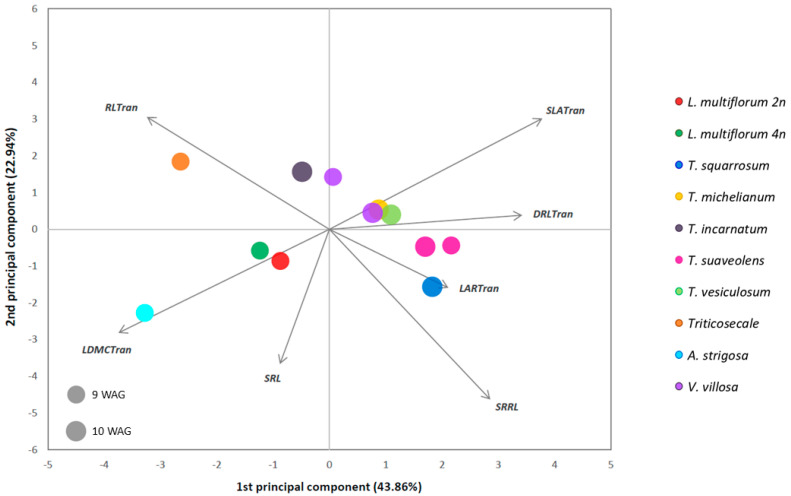
Principal component analysis of morphological data in 10 different annual forage species (grasses and legumes) 9 and/or 10 weeks after germination (WAG). The two principal components accounted for 66.80% of the total variation. DRL—dry root weight/root length ratio, LAR—leaf area, LDMC—leaf dry matter content, RL—root length, SLA—specific leaf area, SRL—secondary root length, SRRL—secondary root length to root length ratio. The suffix “Tran” next to the traits’ abbreviation means that the trait values were transformed.

At the final time points of measurement (9 or 10 WAG), specific leaf area (SLA) and leaf dry matter content (LDMC) (and dry root weight/root length ratio (DRL) to a lesser extent) also allowed for the discrimination between grass and legume species, with grasses having a higher LDMC and a smaller SLA (Figure 2 and Supplementary Table S3). The root and secondary root length did not differentiate between the two families, although diversity was observed within each family. Nonetheless, independently of the family, some species performance was unique for some traits, e.g., *Triticosecale* located in the PCA plot along the vector towards longer main root length, and in the opposite direction, *T. suaveolens* and *T. squarrosum* located along the vector towards a higher secondary root length to root length ratio (Figure 2).

Additionally, *T. squarrosum* was the legume species with the highest leaf area (LAR), and *T. incarnatum* the one with the highest root length (RL), at 10 WAG. Among the grass species, at 9 WAG, *Triticosecale* had the smallest secondary root length to root length ratio (SRRL) and smallest LAR. The *A. strigosa* showed, on the other hand, the highest LDMC, the highest secondary root length (SRL), the smallest SLA, and the smallest DRL.

### 2.2. Photosynthetic Performance under Contrasting Water Regimes

The photosynthetic response of the same 10 grass and legume species was evaluated under well-watered (WW) and water deficit (WD) conditions, with WD being defined as 50% of field capacity, at approximately 7 WAG. Gas-exchange and chlorophyll a fluorescence-related parameters and photosynthetic pigment content were analyzed under both water treatments.

**Figure 3:**
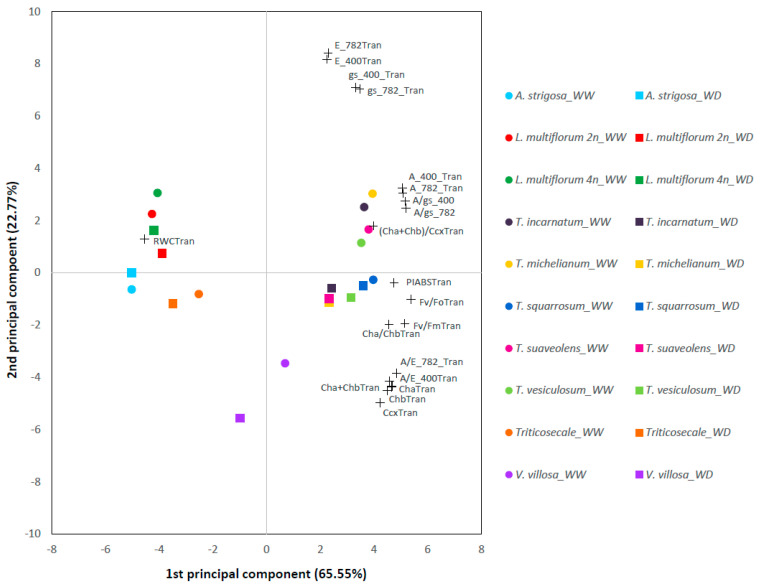
Principal component analysis of photosynthesis-related traits measured in 10 different annual forage species (grasses and legumes), under well-watered (WW, circles) and water deficit conditions (WD, squares). The two principal components accounted for 88.32% of the total variation. Trait’s vectors are represented by a “+” symbol. Trait’s abbreviations: A_400: CO_2_ assimilation rate measured at 400 µmol m^−2^ s^−1^ PAR; A_782: CO_2_ assimilation rate measured at 782 µmol m^−2^ s^−1^ PAR; Cha: chlorophyll a content; Chb: chlorophyll b content; Ccx: total carotenoids content; E_400: transpiration rate measured at 400 µmol m^−2^ s^−1^ PAR; E_782: transpiration rate measured at 782 µmol m^−2^ s^−1^ PAR; Fv/Fm: maximum quantum yield of photochemistry in photosystem II; Fv/Fo: maximum quantum yield of photochemistry in photosystem II normalized by the minimum fluorescence; gs_400: stomatal conductance measured at 400 µmol m^−2^ s^−1^ PAR; gs_782: stomatal conductance measured at 782 µmol m^−2^ s^−1^ PAR, PIABS: performance index; RWC: leaf relative water content. A/E: instantaneous water use efficiency (WUE); A/gs: intrinsic water use efficiency (WUEi). All the pigment contents were expressed in mg per g of dry weight. The suffix “Tran” next to the traits’ abbreviation means that the trait values were transformed.

Significant differences were detected among species for all evaluated traits, regardless of treatment (Supplementary Table S4). The *Trifolium* species had a higher CO_2_ assimilation rate (A), instantaneous water use efficiency WUE (A/E), maximum quantum yield of photochemistry in photosystem II (Fv/Fm), maximum quantum yield of photochemistry in photosystem II normalized by the minimum fluorescence (Fv/Fo), and performance index (PIABS) than all the grass species, independently of the water treatment. The legume species also had higher chlorophyll a and b levels (Cha and Chb) and total carotenoid (Ccx) contents than the grass species not only under WW conditions but also under the tested WD conditions. Nevertheless, among the legumes, *V. villosa* had in general higher chlorophyll pigment contents than the *Trifolium* spp. across treatments. On the other hand, the grass species had higher leaf relative water content (RWC) than the legume species in both water treatments (Supplementary Table S5, Figure 3). Under WW conditions, the *Trifolium* spp. had the highest values of stomatal conductance (gs), while under WD the differences between species were not as clear (Table S5). Interestingly, A, transpiration rate (E), stomatal conductance (gs), and intrinsic water use efficiency WUEi (A/gs) were the traits that best discriminated within species and treatment.

The water-deficit treatment did not result in significant differences in Cha, Chb, Ccx, Cha/Chb, Cha + Chb, or (Cha + Chb/Ccx). Additionally, no significant differences between treatments were observed for Fv/Fm, Fv/Fo, PIABS, or RWC (Supplementary Table S4). Nevertheless, the leaf RWC, the photosynthetic pigments, and the chlorophyll a fluorescence-related parameters (PIABS, Fv/Fm, and Fv/Fo) were the traits that most differentiated the response of the grass and legumes species under both water treatments (Figure 3). Plants’ photosynthetic response was affected by the light intensity, with the values of A, E, and gs being higher at the species’ saturating light than at the typical autumn light intensity in Portugal (782 vs. 400 µmol m^−2^ s^−1^ PAR) in both water treatments. Additionally, there was a strong positive correlation between the gas exchange parameters measured at both light intensities.

## 3. Discussion

Mediterranean annual forage mixtures nowadays face the impact of climate change constraints, especially with increased temperatures and lower water availability. To improve forage mixtures’ performance under drought, there is a need, as a first step, to target the most informative/useful resilience traits to complement the traditionally used selection criteria. It is also important to identify the most drought resilient individual species with more potential for evaluation in a second step in forage mixtures’ combinations. In this context, this study characterized and compared, in higher detail than normally used in forage breeding, below and above ground morphological traits across the first development weeks and the photosynthetic performance under water deficit of representatives of the legume and grass species typically used on Mediterranean annual forage mixtures.

We followed a trait-based approach with measurements from the 3rd or 4th until the 9th or 10th WAG, targeting root and leaf characteristics that might enhance yield stabilization and maximization. For that, we individually analyzed 10 species from the Fabaceae and Poaceae families, well adapted to Portuguese Mediterranean environmental conditions. These species had different growth architectures and thus, were the preferential choice to evaluate the potential and complementarity of the selected traits. Furthermore, we evaluated photosynthesis-related traits under WW and WD conditions in the same species and also at the individual plant level.

This allowed us to rank the most resilient species to water deficit conditions and identify new breeding targets (like photosynthesis-related or below-ground plant traits) that, although not commonly used in forage selection, might explain the observed higher resilience. These traits should support/complement the future optimization of annual forage mixtures to cope with water constraints. This will be particularly important for the vulnerable Mediterranean basin, more prone to severe drought periods.

The quantification of gas exchange parameters and leaf photosynthetic pigments provided another view on the plants’ physiological response under contrasting water regimes. When focusing on photosynthetic performance (CO_2_ assimilation rate, A), *Trifolium michelianum* and *T. incarnatum* were the less resilient species analyzed under WD versus WW conditions, followed closely by *T. suaveolens* and *T. vesiculosum*. Nevertheless, the two first mentioned species were also the best performing species under WW. In fact, among the studied clovers, these two species were only surpassed in WD by the performance of *T. squarrosum*, the most resilient species found. Interestingly, the species following *T. squarrosum* in terms of photosynthetic resilience to WD were *A. strigosa* and *Triticosecale* (smallest differences of A between WW and WD). However, these species were not among the best performing ones under WW nor under WD conditions, only performing better than *V. villosa*, the species with the lowest A values under the tested conditions. Additionally, the leaf photosynthetic pigments content was higher for the legume than for the grass species regardless of the water treatment.

Taking into consideration the detailed above and belowground morphological plant characterization, with a focus on the 7th WAG (the same time point used for the water deficit experiment), a diversity of potential resilience approaches was identified among the studied annual forage mixture individual components. Yield stabilization and maximization of forage mixtures are typically related to root and aerial architectures, plant length, tillering, canopy height, and biomass [16]. The species previously detected as the most resilient to water deficit, *T. squarrosum*, *A. strigosa*, and *Triticosecale*, were among the ones with the highest root to shoot ratio (R/S). *Trifolium squarrosum* was also highlighted due to its high secondary root length to root length ratio (SRRL) and root weight to root length ratio (DRL), measured at 10 WAG. Interestingly, *A. strigosa* and *Triticosecale* were among the species with the lowest values of these two root traits. Thus, these two species might have additional but different traits contributing to their behavior under water deficit. It has also been described that, depending on the species, plants may exhibit some plasticity in root traits, namely in the ones related to root density, such as DRL, under a water scarcity situation [14]. *Triticosecale* was the species with the longest root and the smallest leaf area. Interestingly, in the water-deficit response experiment, *Triticosecale* was the grass species with higher WUE, a trait that is known to be associated with lower SLA as a strategy of phenotypic adjustment [17]. On the other hand, *A. strigosa* was the species with the highest canopy height, R/S, and leaf dry matter content (LDMC). High LDMC has been described to be characteristic of drought-tolerant annual grassland species [18]. Root depth and growth are key traits in plants’ adaptation to drought tolerance [19] and the root development observed in the grass species is potentially a strategy that might confer an advantage under water scarce conditions. Indeed, to acquire and retain enough water to maintain growth under drought conditions, plants may allocate more mass to roots through the ability to elongate roots into deeper soil layers and form thin and highly branched root systems [20].

Besides *Triticosecale*, *L. multiflorum* 2n and 4n, *V. villosa*, and *T. incarnatum* were also among the species with the highest root lengths. On the other hand, along with *T. squarrosum*, *T. suaveolens*, and *T. vesiculosum* also had high SRRL and DRL. Moreover, *T. incarnatum* was characterized by the longest roots of all the legumes. This species, along with *T. michelianum*, although considered less resilient considering the photosynthetic response under WD versus WW conditions, were the best performing species under WW conditions. Indeed, *T. michelianum* was one of the species with the biggest aerial but also root dry weights, being surpassed only by *V. villosa*, but with a corresponding smaller root to shoot ratio. *Vicia villosa*, although not among the most resilient to WD, was the species with the fastest early development and the highest plant length at the end of the experiment.

*T. vesiculosum* had a similar development pattern to *T. michelianum*, but was smaller by the end of the experiment. This species was similar in final aerial size and WD resilience to *T. suaveolens*, but the latter was clearly differentiated by its higher RDW and corresponding higher R/S. Among the grass species, the two *L. multiflorum* under analysis (2n and 4n) behaved similarly in what concerned WD resilience, but the 4n species had a faster and higher development and overall better performance. Besides the diversity of WD resilience strategies/approaches identified among the annual forage mixtures’ individual components, it was also possible to identify several complementarities between the grass and legume species evaluated. These findings suggest the potential of mixing species from both families with different strategies to achieve good performances and attain high WD resilience. Overall, we found that the photosynthetic response of the grass species is inferior to the one of legumes species under both water treatments. Nevertheless, the grass species showed higher values of canopy height and R/S than the legume species. Additionally, grasses presented faster early development (at 3–4 WAG), but as the development progressed, the legume species, and in particular *V. villosa* and *T. michelianum*, overcame the grasses development with a much higher aerial and root dry biomass (as at 9 WAG). However, around this time point, the grass species presented, in general, a higher leaf dry matter content, and an associated smaller specific leaf area. Complementary plant architecture can bring advantages in the caption of solar energy for photosynthesis [21]. The plant growth habit, leaf arrangement, leaf angle, or branching strongly influence the interception of light and the radiation partitioning, which can lead to a complementary use of light by plant mixtures [22]. In fact, as already pointed out with the R/S differences, legume and grass species prioritized shoot or root development in opposite ways. Legumes invested more in the shoot development whereas grasses privileged the root more. This investment in the root system might explain why leaf RWC and LDMC were higher in grasses than in legumes. It was described that under water scarcity, forage plants are able to acquire enough soil water while minimizing evapotranspiration through a higher root/shoot ratio [23].

In the WW vs. WD experiment, the legume species had higher values of all the photosynthesis-related parameters, namely, Cha, Chb, Ccx, A, E, gs, WUE, Fv/Fm and PIABS, than the grass species. This might be indicative of the legumes’ strategy when facing a water deficit being more concentrated in the plant aerial fraction than in the roots, in accordance also with their smaller R/S. Within the legumes, all the clovers had better photosynthetic performance than *V. villosa*.

The specificities and complementarity of responses observed in grass and legume species suggest that a mixture of selected resilient species/genotypes from both families should enhance annual forage productivity through increased resilience and plasticity. One limitation of our work was, however, that we only evaluated one genotype of each species, hampering the assessment of intraspecific variability. This assessment will be fundamental to advance species’ mixtures optimization. A follow-up of this study could take advantage of the work described here and evaluate different varieties of each species using the most differentiating traits identified.

Species biodiversity influences the way plant communities respond to climate changes or environmental instabilities [6,24], with an effect on plant productivity [25]. In grassland experiments, species grown in mixtures tend to develop contrasting trait values through strong complementarity effects [26] that must be potentiated. Nevertheless, trade-offs between functional characteristics may influence the agronomic and ecological success of mixtures of varieties [16]. Additionally, the nature of traits to be selected depends on agricultural scenarios. The characterization of individual plants described in the present study must now progress to the use of the most relevant traits directly in plant mixtures.

In plant mixtures, the different species interact and compete for nutrients, water, and sun light. For example, nutrient competition might lead to higher root length, and light competition to taller plants with flatter canopies, than would be the case in the absence of competition [27]. Furthermore, complex interactions between physiological and genetic factors involved in plant mixtures may also cause trade-offs, or negative correlations, between traits in processes still difficult to define [28].

Next steps will include evaluating the same functional traits in different mixtures composed of these species under controlled and field conditions to assess the plants’ interrelationships and complementarities as mixtures. For example, under water-deficit conditions, the water absorption capacity of roots or leaf gas exchange regulation by stomata are traits, although more laborious, that should now be prioritized also in mixture evaluation.

To conclude, the present study establishes a framework of useful traits to be included in the selection criteria and interesting sources for future annual forage mixture breeding for climate change. We found that the highest canopy height and root to shoot ratio of the grass species can be well complemented with the higher aerial and root biomass and superior photosynthetic performance of the legume species. We highlight *T. squarrosum* and *Triticosecale*, a legume and a grass, respectively, as the two species from the typical grass–legume mixture components tested with the best and most resilient photosynthetic response under the two water treatments applied.

The specificities, complementarities, and potential beneficial interactions among legume and grass species identified will contribute to the development of innovative annual forage mixtures, making them more resilient and productive to help overcome climate change’s negative impact and promote more sustainable agriculture.

## 4. Materials and Methods

### 4.1. Plant Material and Growth Conditions

Seeds from the grass species *Avena strigosa*, *Lolium multiflorum* 2n, *L. multiflorum* 4n, and *Triticosecale* (Poaceae) and from the legume species *Trifolium incarnatum*, *T. michelianum*, *T. squarrosum*, *T. suaveolens*, *T.vesiculosum*, and *Vicia villosa* (Fabaceae) were sown in 8 cm × 8 cm × 9 cm plastic pots (0.5 L) filled with Montemor soil/peat/vermiculite (2:1:1 (*v*/*v*)) (one seed per pot). These species are components of biodiverse annual forage mixtures rich in legumes (normally composed of 5–7 different species) and adapted to different soil and climate conditions of annual grassland pasture and forage production areas. These mixtures were developed by Fertiprado, a Portuguese seed company, and were under study in the scope of the Horizon 2020 DIVERSify project (https://plant-teams.org, accessed on 31 July 2021).

Since under the Portuguese agricultural systems the studied species are sown in autumn, pots were placed in growth chambers under similar environmental conditions (temperature 20 °C day/14 °C night; photoperiod 11 h; relative humidity 60%; light intensity ~400 µmol m^−2^ s^−1^).

### 4.2. Species Morphological Development Characterization Experiment

Two growth chambers (3.30 m × 1.60 m) were used in parallel to accommodate all the plants (Figure S1), grouping species according to their development speed. *Avena strigosa*, *L. multiflorum* 2n and 4n, *Triticosecale*, *T. suaveolens*, and *V. villosa* were grown in one growth chamber, whereas the slower initial development species *T. incarnatum*, *T. michelianum*, *T. squarrosum*, *T. vesiculosum*, *T. suaveolens*, and *V. villosa* were grown in a second growth chamber. *Trifolium suaveolens* and *V. villosa* plants were used as between-chamber controls. For seven weeks, destructive measurements were taken weekly in an average of 10 plants per species. In the first growth chamber, with the fastest initial development grass species, measurements started at the third week after germination (WAG) and ended at the ninth WAG, while in the second growth chamber, with the legume species, measurements started only at the fourth WAG and ended at the tenth WAG. This allowed for a total of seven weeks of monitoring in all species. This characterization focused on the plant’s first developmental stages, until 9–10 WAG, as the most affected in an autumn–winter drought. In total, 886 individual plants were evaluated in this experiment.

### 4.3. Species Photosynthetic Performance Characterization under Contrasting Water Regimes Experiment

For the species photosynthetic performance characterization experiment, two water treatments were applied to the plants in the same growth chamber (Figure S2). Half of the plants (pots) were subjected to water deficit (WD, corresponding to 50% of field capacity, estimated by weighing the pots), and the other half were kept constantly under well-watered conditions. WD was imposed by watering withdrawal 40 days after germination and measurements took place when 50% of field capacity was reached, approximately 10 days later, at the 7th WAG. Since it was not possible to perform the laborious photosynthetic performance measurements in all plants on the same day, seed germination was done sequentially, and plants were divided into six groups. The measurements were staggered to occur in a different week for each group. At each group, the 10 species (three plants per species and water treatment) were divided over three days: each day with three of the 10 species, plus *T. suaveolens* that was repeatedly evaluated in all the three sampling days per week/group, as controls. In total, 432 individual plants were evaluated (36 plants per species, except for *T. suaveolens* that had triple this many (108) plants evaluated), half under each water treatment.

### 4.4. Morphological and Physiological Measurements

At the morphological development characterization experiment, the weekly measured traits were aerial fraction dry weight (ADW), root fraction dry weight (RDW), canopy height (CH), plant growth habit (PGH), plant length (PL), branching (B, only for legume species), number of root nodules (N, only for legume species), and tillering (T, only for grass species). With the data obtained, dry root to dry shoot ratio (R/S) was also calculated. Extra measurements were taken on the last weeks (9 or 10 WAG) for all species: root length (RL), and secondary root length (SRL), leaf area (LAR), leaf dry weight (DW), leaf fresh weight (FW), and leaf turgid weight (TW). With the data obtained, dry root weight to root length ratio (DRL), specific leaf area (SLA), secondary root length to root length ratio (SRRL), and leaf dry matter content (LDMC) were also calculated.

At the photosynthetic performance experiment, the traits assessed under both water regimes were leaf relative water content (RWC), obtained as RWC (%) = ((FW − DW)/(TW − DW)) × 100; chlorophylls a and b (Cha and Chb), and total carotenoid (Ccx) contents (quantified using a spectrophotometer according to Wintermans and De Mots [29] and expressed in mg per g of leaf dry weight); maximum quantum yield of photochemistry in photosystem II (Fv/Fm), maximum quantum yield of photochemistry in photosystem II normalized by the minimum fluorescence (Fv/Fo), and performance index (PIABS) using a chlorophyll fluorometer (OS30p+, Opti-Sciences, Hudson, NY, USA); and gas-exchange parameters, namely net CO_2_ assimilation rate (A), transpiration rate (E), and stomatal conductance (gs). The gas exchange parameters were measured using an infrared gas analyzer system (IRGA, LCpro+ ADC BioScientific Ltd., Hertfordshire, UK) at two light intensities corresponding to an average autumn light intensity in Portugal (400 µmol m^−2^ s^−1^ photosynthetically active radiation (PAR)) and saturating photosynthetic light (782 PAR) for the species evaluated. A, E, and gs values were used to calculate instantaneous and intrinsic water-use efficiencies (WUE = A/E and WUEi = A/gs, respectively). The sum of Cha and Chb, their ratio, and the ratio between the sum of chlorophylls and carotenes and xanthophylls [(Cha + Chb)/Ccx] were calculated. All the measurements followed the guidelines described in the handbook for trait assessment in agricultural plant teams [30].

### 4.5. Statistical Analyses

Differences between species over time were statistically analyzed using Genstat (Genstat^®^ for Windows 20th edition, VSN International, Hemel Hempstead, UK). Data quality control (outliers and homogeneity of variance), analysis of variance (ANOVA), comparison of means (Tukey’s), and principal component analysis were performed based on linear mixed models adjusted mean values. To stabilize the variance of the traits, the Box-Cox transformation procedure was conducted, and the most appropriate transformation applied.

For the traits evaluated across time, a linear mixed model was fitted per trait as trait = species + WAG + species × WAG + chamber × species using the restricted maximum likelihood (REML) variance component analysis framework of Genstat. The species (one to ten), the number of weeks after germination (WAG, three to ten), and their interaction were treated as fixed effects and species nested within each growth chamber (one or two) as a random effect. For the traits measured only at the last time point, a similar model without the WAG term was used.

For the photosynthesis performance experiment, a linear mixed model was fitted per trait as trait = species + treatment + species × treatment + group × species, with species (one to ten), the treatment (WW or WD), and their interaction treated as fixed effects and species nested within each group (one to six) treated as a random effect.

Those models, with species as a fixed effect, allowed the estimation of the best linear-unbiased estimates (BLUEs) for each species, which were subsequently used to perform a principal component analysis (PCA).

## Data Availability

The data presented in this study are available in FigShare at https://doi.org/10.6084/m9.figshare.16350723.v1 (accessed on 22 August 2021).

## Supplementary Materials

The following are available online at https://www.mdpi.com/article/10.3390/plants10091763/s1, Figures S1 and S2: Schematic representation of the experiments, Table S1: Analysis of variance for the traits measured on 10 annual forage species in each week, Table S2: Average and standard deviation values for the morphological traits measured in 10 different annual forage species 3 to 10 weeks after germination (WAG), Table S3: Average morphological and standard deviation values for 10 annual forage species, measured 9 or 10 weeks after germination (WAG), Table S4: Analysis of variance for the physiological traits measured for 10 annual forage species under two water regimens (well-watered and water deficit), Table S5: Average and standard deviation values for physiological traits measured under well-watered (WW) and water deficit (WD) conditions in 10 annual forage species.
