# Supplementary material for: Towards a Trait-Based Approach to Potentiate Yield under Drought in Legume-Rich Annual Forage Mixtures
Source: Plants (Basel). 2021 Aug 25;10(9):1763. doi: 10.3390/plants10091763 (PMC8467334; doi:10.3390/plants10091763)
Supplement: Supplementary file 1 [file plants-10-01763-s001.zip › plants-1343680-supplementary.pdf]

Supplementary Figure 1

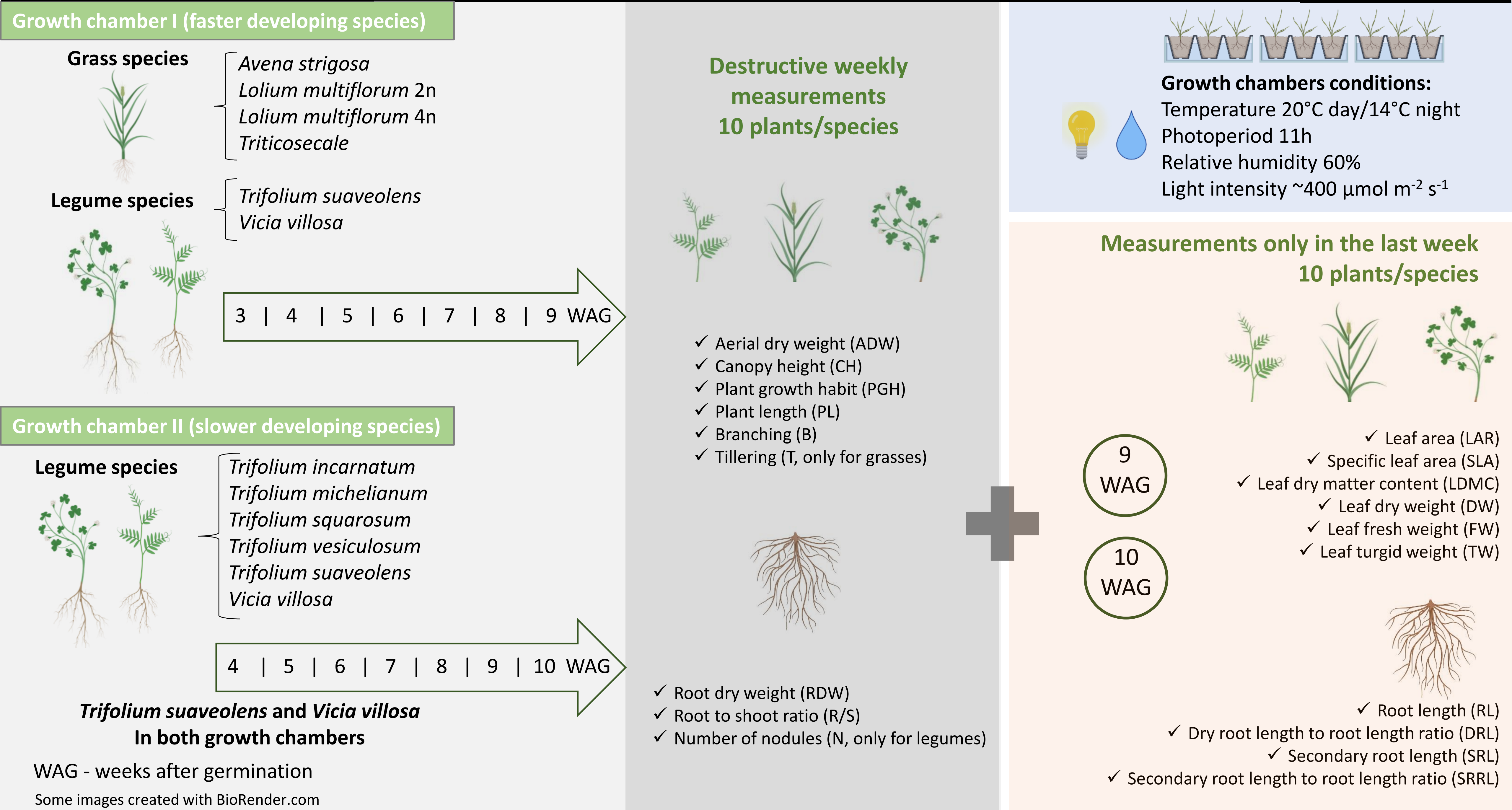

Supplementary Figure 2 **2) Photosynthetic performance under contrasting water regimes experiment**

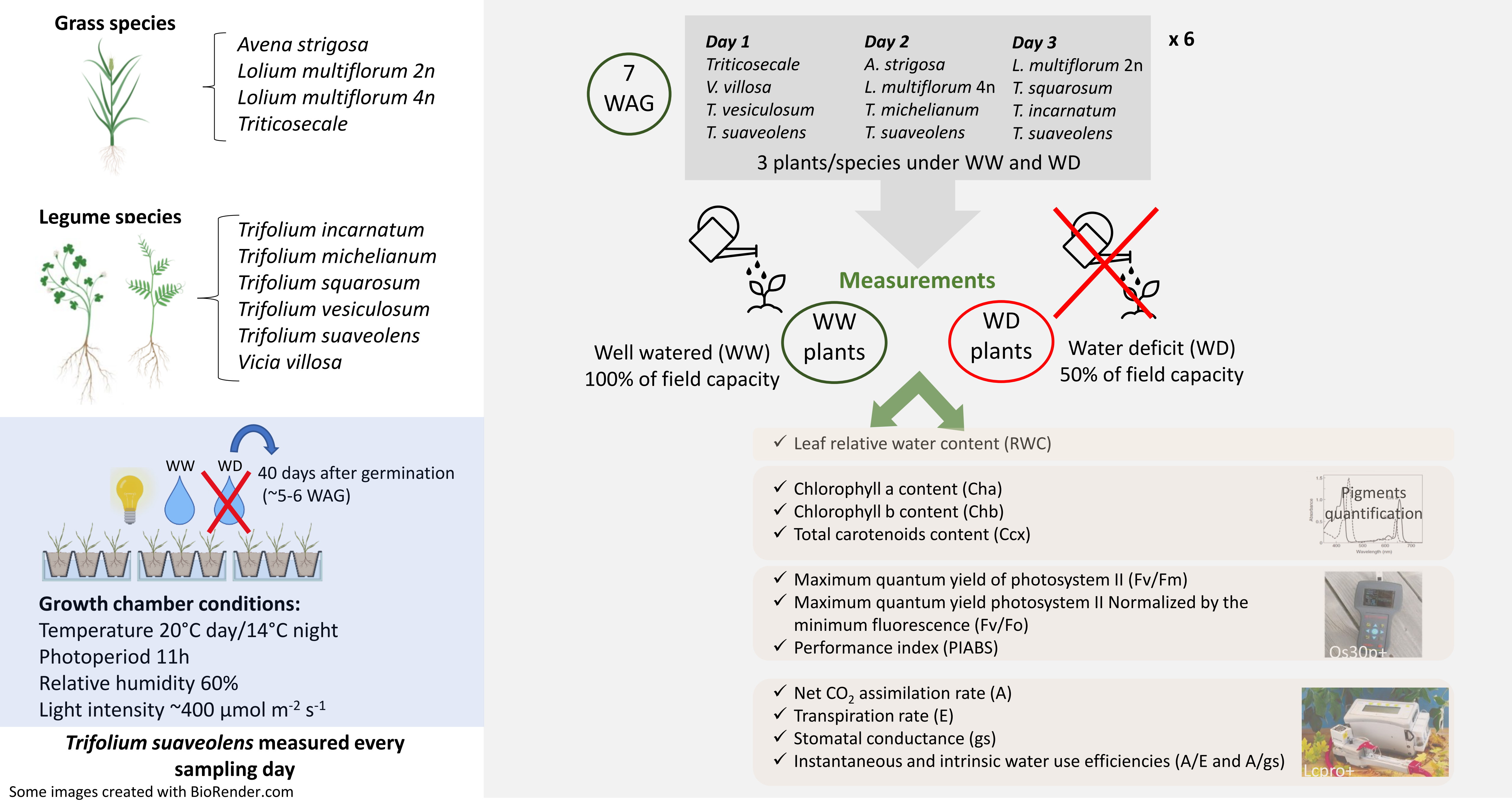

Towards a trait-based approach to potentiate yield under drought in legume-rich annual forage mixtures

Susana T. Leitão, Mara Lisa Alves, Priscila Pereira, Aziza Zerrouk, Bruno Godinho, Ana Barradas, Maria Carlota Vaz Patto

Supplementary Tables

Table S1: Analysis of variance for the traits measured on 10 annual forage species, at each week. WAG – weeks after germination. Traits' abbreviations: ADW - aerial fraction dry weight (g), B – branching, CH - canopy height (cm), DRL - dry root weight/root length ratio (g cm^{-1}), LAR - leaf area (mm^2), LDMC - leaf dry matter content (mg g^{-1}), N - number of root nodules, PGH - plant growth habit, PL - plant length (cm), RDW - root fraction dry weight (g), RL - root length (cm), R/S - dry root to dry shoot ratio, SLA - specific leaf area (mm_2 mg^{-1}), SRL - secondary root length (cm), SRRL - secondary root length to root length ratio, T – tillering.

Trait	WAG	Source of variation	d.f.	s.s.	m.s.	v.r.	F pr.
ADW (Box-Cox transformation of ADW on logarithmic scale)	3	Species	5	106.287	21.257	121.131	<.001
		Residual	57	10.003	0.175		
		Total	62	98.627			
ADW (Box-Cox transformation of ADW on logarithmic scale)	4	Species	9	119.832	13.315	125.073	<.001
		Residual	107	11.391	0.106		
		Total	116	114.318			
ADW (Box-Cox transformation of ADW on logarithmic scale)	5	Species	9	94.752	10.528	75.644	<.001
		Residual	110	15.310	0.139		
		Total	119	96.559			
ADW (Box-Cox transformation of ADW on logarithmic scale)	6	Species	9	83.188	9.243	43.019	<.001
		Residual	113	24.279	0.215		
		Total	122	96.537			
ADW (Box-Cox transformation of ADW on logarithmic scale)	7	Species	9	82.108	9.123	39.076	<.001
		Residual	113	26.382	0.233		
		Total	122	97.302			
ADW (Box-Cox transformation of	8	Species	9	119.334	13.259	42.591	<.001

ADW on logarithmic scale)		Residual	119	37.047	0.311		
		Total	128	143.873			
ADW (Box-Cox transformation of ADW on logarithmic scale)	9	Species	9	159.201	17.689	98.015	<.001
		Residual	126	22.739	0.180		
		Total	135	177.102			
ADW (Box-Cox transformation of ADW on logarithmic scale)	10	Species	5	10.716	2.143	9.341	<.001
		Residual	56	12.849	0.229		
		Total	61	23.489			
B	3	Species	1	4.351	4.351	15.437	<.001
		Residual	20	5.636	0.282		
		Total	21	9.318			
B	4	Species	5	19.645	3.929	9.770	<.001
		Residual	72	28.955	0.402		
		Total	77	46.218			
B	5	Species	5	110.228	22.046	30.643	<.001
		Residual	74	53.238	0.719		
		Total	79	148.200			
B	6	Species	5	230.353	46.071	58.198	<.001
		Residual	77	60.955	0.792		
		Total	82	268.265			
B	7	Species	5	417.765	83.553	66.432	<.001
		Residual	77	96.844	1.258		
		Total	82	473.060			
B	8	Species	5	823.291	164.658	82.728	<.001
		Residual	74	147.286	1.990		
		Total	79	822.750			
B	9	Species	5	831.898	166.380	101.648	<.001

		Residual	80	130.946	1.637		
		Total	85	899.256			
		Species	5	89.804	51.402	33.480	<.001
B	10	Residual	57	1.747			
		Total	62				
		Species	5	2963.713	592.743	136.368	<.001
CH	3	Residual	57	247.759	4.347		
		Total	62	2787.459			
	4	Species	9	6479.314	719.924	222.395	<.001
CH		Residual	109	352.848	3.237		
		Total	118	5865.098			
	5	Species	9	5155.743	572.860	98.045	<.001
CH		Residual	112	654.395	5.843		
		Total	121	5310.239			
	6	Species	9	7245.918	805.102	131.730	<.001
CH		Residual	114	696.740	6.112		
		Total	123	7242.440			
	7	Species	9	8536.036	948.448	134.230	<.001
CH		Residual	112	791.374	7.066		
		Total	121	8258.641			
	8	Species	9	9489.296	1054.366	166.494	<.001
CH		Residual	118	747.265	6.333		
		Total	127	9525.722			
	9	Species	9	9736.560	1081.840	186.585	<.001
CH		Residual	123	713.169	5.798		
		Total	132	9948.384			
CH	10	Species	5	879.026	175.805	35.159	<.001

		Residual	56	280.014	5.000		
		Total	61	1100.874			
DRL (Box-Cox transformation of DRL on logarithmic scale)	9	Species	5	13.838	2.768	19.165	<.001
		Residual	67	9.675	0.144		
		Total	72	19.309			
DRL (Box-Cox transformation of DRL on logarithmic scale)	10	Species	5	3.804	0.761	5.203	<.001
		Residual	56	8.188	0.146		
		Total	61	11.890			
LAR (Box-Cox transformation of LAR with parameter −0.25)	9	Species	5	0.915	0.183	91.237	<.001
		Residual	65	0.130	0.002		
		Total	70	0.780			
LAR (Box-Cox transformation of LAR with parameter −0.25)	10	Species	5	0.548	0.110	66.323	<.001
		Residual	56	0.093	0.002		
		Total	61	0.622			
LDMC (Box-Cox transformation of LDMC with parameter −0.75)	9	Species	5	0.005	0.001	89.626	<.001
		Residual	65	0.001	0.000		
		Total	70	0.004			
LDMC (Box-Cox transformation of LDMC with parameter −0.75)	10	Species	5	0.001	0.000	9.787	<.001
		Residual	56	0.001	0.000		
		Total	61	0.001			
N (Box-Cox transformation of N*+0.5* with parameter 0.25)	3	Species	1	95.394	95.394	45.797	<.001
		Residual	20	41.660	2.083		
		Total	21	122.379			
N (Box-Cox transformation of N*+0.5* with parameter 0.25)	4	Species	5	170.906	34.181	16.336	<.001
		Residual	72	150.651	2.092		
		Total	77	297.444			
N (Box-Cox transformation of N*+0.5*	5	Species	5	115.678	23.136	26.478	<.001

with parameter 0.25)		Residual	75	65.534	0.874		
		Total	80	168.577			
N (Box-Cox transformation of N*+0.5* with parameter 0.25)	6	Species	5	320.142	64.028	54.866	<.001
		Residual	77	89.858	1.167		
		Total	82	382.404			
N (Box-Cox transformation of N*+0.5* with parameter 0.25)	7	Species	5	201.748	40.350	24.474	<.001
		Residual	76	125.297	1.649		
		Total	81	307.151			
N (Box-Cox transformation of N*+0.5* with parameter 0.25)	8	Species	5	420.810	84.162	29.423	<.001
		Residual	76	217.395	2.860		
		Total	81	609.042			
N (Box-Cox transformation of N*+0.5* with parameter 0.25)	9	Species	5	324.735	64.947	14.951	<.001
		Residual	77	334.487	4.344		
		Total	82	636.683			
N (Box-Cox transformation of N*+0.5* with parameter 0.25)	10	Species	5	133.071	26.614	23.112	<.001
		Residual	57	65.638	1.152		
		Total	62	198.709			
PGH	4	Species	5	245.640	49.128	167.023	<.001
		Residual	51	15.001	0.294		
		Total	56	136.561			
PGH	5	Species	5	188.585	37.717	106.261	<.001
		Residual	51	18.102	0.355		
		Total	56	121.895			
PGH	6	Species	5	123.811	24.762	38.661	<.001
		Residual	55	35.227	0.640		
		Total	60	104.590			
PGH	7	Species	5	137.876	27.575	44.178	<.001

		Residual	55	34.330	0.624		
		Total	60	109.128			
PGH	8	Species	5	134.442	26.888	52.147	<.001
		Residual	54	27.844	0.516		
		Total	59	115.246			
PGH	9	Species	5	112.361	22.472	36.468	<.001
		Residual	57	35.124	0.616		
		Total	62	93.913			
PGH	10	Species	5	52.309	10.462	27.950	<.001
		Residual	56	20.961	0.374		
		Total	61	72.919			
PI_{ABS}	10	Species	4	2893.761	723.440	5.133	0.002
		Residual	45	6342.151	140.937		
		Total	49	9174.271			
PL (Box-Cox transformation of PL on logarithmic scale)	3	Species	5	174.058	34.812	164.290	<.001
		Residual	57	12.078	0.212		
		Total	62	158.746			
PL (Box-Cox transformation of PL on logarithmic scale)	4	Species	9	361.389	40.154	236.253	<.001
		Residual	106	18.016	0.170		
		Total	115	328.922			
PL (Box-Cox transformation of PL on logarithmic scale)	5	Species	9	351.005	39.001	239.027	<.001
		Residual	107	17.459	0.163		
		Total	116	322.205			
PL (Box-Cox transformation of PL on logarithmic scale)	6	Species	9	304.910	33.879	486.267	<.001
		Residual	113	7.873	0.070		
		Total	122	283.393			
PL (Box-Cox transformation of PL on	7	Species	9	323.821	35.980	330.475	<.001

logarithmic scale)		Residual	112	12.194	0.109		
		Total	121	302.409			
PL (Box-Cox transformation of PL on logarithmic scale)	8	Species	9	295.318	32.813	308.902	<.001
		Residual	118	12.535	0.106		
		Total	127	289.513			
PL (Box-Cox transformation of PL on logarithmic scale)	9	Species	9	286.075	31.786	334.892	<.001
		Residual	126	11.959	0.095		
		Total	135	297.972			
PL (Box-Cox transformation of PL on logarithmic scale)	10	Species	5	136.281	27.256	682.958	<.001
		Residual	57	2.275	0.040		
		Total	62	138.556			
R/S (Box-Cox transformation of R/S*+0.5* with parameter -1)	3	Species	5	2.47940	0.49588	7.91	<.001
		Residual	58	3.63594	0.06269		
		Total	63	5.80088			
R/S (Box-Cox transformation of R/S*+0.5* with parameter -1)	4	Species	9	7.37092	0.81899	22.51	<.001
		Residual	108	3.92910	0.03638		
		Total	117	9.99242			
R/S (Box-Cox transformation of R/S*+0.5* with parameter -1)	5	Species	9	8.74036	0.97115	48.64	<.001
		Residual	112	2.23628	0.01997		
		Total	121	9.88512			
R/S (Box-Cox transformation of R/S*+0.5* with parameter -1)	6	Species	9	7.36299	0.81811	61.90	<.001
		Residual	113	1.49341	0.01322		
		Total	122	8.11760			
R/S (Box-Cox transformation of R/S*+0.5* with parameter -1)	7	Species	9	9.95322	1.10591	49.85	<.001
		Residual	111	2.46246	0.02218		
		Total	120	11.15101			

R/S (Box-Cox transformation of R/S+*0.5* with parameter -1)	8	Species	9	11.44821	1.27202	85.59	<.001
		Residual	119	1.76862	0.01486		
		Total	128	12.04337			
R/S (Box-Cox transformation of R/S+*0.5* with parameter -1)	9	Species	9	15.41669	1.71297	114.27	<.001
		Residual	126	1.88876	0.01499		
		Total	135	16.81418			
R/S (Box-Cox transformation of R/S+*0.5* with parameter -1)	10	Species	5	0.78414	0.15683	11.37	<.001
		Residual	57	0.78602	0.01379		
		Total	62	1.57016			
RDW (Box-Cox transformation of RDW with parameter 0.25)	3	Species	5	10.642	2.128	143.827	<.001
		Residual	55	0.814	0.015		
		Total	60	9.439			
RDW (Box-Cox transformation of RDW with parameter 0.25)	4	Species	9	19.946	2.216	174.993	<.001
		Residual	108	1.368	0.013		
		Total	117	18.544			
RDW (Box-Cox transformation of RDW with parameter 0.25)	5	Species	9	20.245	2.249	82.997	<.001
		Residual	110	2.981	0.027		
		Total	119	21.007			
RDW (Box-Cox transformation of RDW with parameter 0.25)	6	Species	9	21.479	2.387	67.371	<.001
		Residual	113	4.003	0.035		
		Total	122	23.543			
RDW (Box-Cox transformation of RDW with parameter 0.25)	7	Species	9	14.298	1.589	37.858	<.001
		Residual	113	4.742	0.042		
		Total	122	17.828			
RDW (Box-Cox transformation of RDW with parameter 0.25)	8	Species	9	15.483	1.720	25.155	<.001
		Residual	119	8.138	0.068		

		Total	128	22.677			
RDW (Box-Cox transformation of RDW with parameter 0.25)	9	Species	9	11.299	1.255	30.968	<.001
		Residual	125	5.067	0.041		
		Total	134	15.968			
RDW (Box-Cox transformation of RDW with parameter 0.25)	10	Species	5	1.768	0.354	4.060	0.003
		Residual	57	4.965	0.087		
		Total	62	6.733			
RL (Box-Cox transformation of RL on logarithmic scale)	9	Species	5	7.370	1.474	15.389	<.001
		Residual	66	6.322	0.096		
		Total	71	11.481			
RL (Box-Cox transformation of RL on logarithmic scale)	10	Species	5	1.480	0.296	3.237	0.010
		Residual	57	5.211	0.091		
		Total	62	6.691			
SLA (Box-Cox transformation of SLA with parameter 0.75)	9	Species	5	3882.945	776.589	132.066	<.001
		Residual	64	376.340	5.880		
		Total	69	3018.354			
SLA (Box-Cox transformation of SLA with parameter 0.75)	10	Species	5	325.345	65.069	3.996	0.004
		Residual	55	895.492	16.282		
		Total	60	1209.861			
SRL (Box-Cox transformation of SRL on logarithmic scale)	9	Species	2	0.445	0.223	7.014	0.003
		Residual	33	1.048	0.032		
		Total	35	1.493			
SRL (Box-Cox transformation of SRL on logarithmic scale)	10	Species	5	1.016	0.203	10.136	<.001
		Residual	57	1.143	0.020		
		Total	62	2.159			
SRRL	9	Species	5	0.663	0.133	10.823	<.001
		Residual	65	0.796	0.012		

		Total	70	1.298			
SRRL	10	Species	5	0.369	0.074	5.858	<.001
		Residual	57	0.717	0.013		
		Total	62	1.086			
T	3	Species	3	0.000			
		Residual	38	0.000			
		Total	41	0.000			
T	4	Species	3	0.000			
		Residual	37	0.000			
		Total	40	0.000			
T	5	Species	3	0.000			
		Residual	35	0.000			
		Total	38	0.000			
T	6	Species	3	0.000			
		Residual	37	0.000			
		Total	40	0.000			
T	7	Species	3	0.000			
		Residual	35	0.000			
		Total	38	0.000			
T	8	Species	3	0.280	0.093	2.127	0.113
		Residual	38	1.667	0.044		
		Total	41	1.905			
T	9	Species	3	0.000			
		Residual	41	0.000			
		Total	44	0.000			

Table S2: Average and standard deviations values for the morphological traits measured in 10 different annual forage species, 3 to 10 weeks after germination (WAG). For each time point, the same letter in columns indicates that the differences among values are not statistically significant ($P < 0.05$, Tukey´s test). Traits' abbreviations: ADW - aerial fraction dry weight (g), B – branching, CH - canopy height (cm), N - number of root nodules, PGH - plant growth habit, PL - plant length (cm), RDW - root fraction dry weight (g), R/S - dry root to dry shoot ratio, T – tillering.

Species	WAG	ADW				CH				PL				RDW			
A. strigosa	3	0.015	±	0.004	c	17.830	±	2.581	a	2.627	±	0.914	b	0.021	±	0.006	b
L. multiflorum 2n		0.005	±	0.001	d	10.930	±	1.679	b	1.056	±	0.553	c	0.003	±	0.001	d
L. multiflorum 4n		0.007	±	0.002	d	11.790	±	2.926	b	1.309	±	0.624	c	0.005	±	0.002	c
T. suaveolens		0.003	±	0.001	e	1.836	±	0.702	c	0.245	±	0.151	d	0.010	±	0.029	e
Triticosecale		0.062	±	0.063	b	17.430	±	2.590	a	3.827	±	0.833	b	0.038	±	0.016	a
V. villosa		0.067	±	0.015	a	2.927	±	0.873	c	27.060	±	2.452	a	0.026	±	0.007	ab
A. strigosa	4	0.059	±	0.079	c	18.890	±	2.585	a	4.109	±	0.722	b	0.052	±	0.015	a
L. multiflorum 2n		0.012	±	0.001	ef	16.200	±	2.024	bc	2.378	±	0.349	b	0.010	±	0.002	d
L. multiflorum 4n		0.020	±	0.005	de	15.770	±	2.291	c	2.773	±	0.471	b	0.018	±	0.003	c
T. incarnatum		0.020	±	0.005	cd	3.736	±	0.987	de	0.527	±	0.241	c	0.009	±	0.003	d
T. michelianum		0.007	±	0.002	g	3.040	±	0.793	de	0.470	±	0.195	c	0.003	±	0.001	e
T. squarrosum		0.008	±	0.002	fg	1.680	±	0.820	de	0.300	±	0.245	d	0.005	±	0.001	e
T. suaveolens		0.007	±	0.003	g	3.642	±	1.001	de	0.432	±	0.216	c	0.004	±	0.001	e
T. vesiculosum		0.005	±	0.004	h	1.467	±	1.041	e	0.300	±	0.190	d	0.001	±	0.001	f
Triticosecale		0.039	±	0.009	b	18.460	±	3.355	ab	3.700	±	0.852	b	0.053	±	0.015	a
V. villosa		0.077	±	0.027	a	3.832	±	1.612	d	27.840	±	5.537	a	0.034	±	0.009	b
A. strigosa	5	0.037	±	0.021	c	20.390	±	4.283	a	3.342	±	0.605	bc	0.057	±	0.020	b
L. multiflorum 2n		0.013	±	0.003	cd	13.160	±	2.352	b	1.887	±	0.562	d	0.025	±	0.038	d
L. multiflorum 4n		0.028	±	0.007	e	11.150	±	3.744	b	2.618	±	0.613	cd	0.031	±	0.006	c
T. incarnatum		0.033	±	0.003	c	4.436	±	0.524	c	0.427	±	0.190	fg	0.014	±	0.002	d
T. michelianum		0.026	±	0.011	cd	4.890	±	1.670	c	0.440	±	0.310	gh	0.010	±	0.004	d

T. squarrosum		0.018	±	0.004	de	2.930	±	0.673	c	0.910	±	0.260	e	0.011	±	0.002	d
T. suaveolens		0.028	±	0.042	e	5.829	±	2.410	c	0.586	±	0.357	ef	0.013	±	0.017	d
T. vesiculosum		0.016	±	0.005	e	4.086	±	0.743	c	0.271	±	0.206	h	0.007	±	0.003	d
Triticosecale		0.063	±	0.011	b	20.400	±	2.913	a	4.591	±	0.541	b	0.109	±	0.054	a
V. villosa		0.171	±	0.094	a	4.318	±	1.630	c	34.750	±	7.746	a	0.065	±	0.023	b
A. strigosa	6	0.066	±	0.021	bc	25.120	±	3.168	a	4.245	±	0.731	bc	0.117	±	0.024	a
L. multiflorum 2n		0.033	±	0.017	d	16.270	±	3.343	c	2.425	±	0.858	d	0.032	±	0.019	bc
L. multiflorum 4n		0.038	±	0.014	cd	15.530	±	3.975	c	3.073	±	0.595	cd	0.040	±	0.013	b
T. incarnatum		0.047	±	0.012	bcd	3.873	±	0.542	e	0.545	±	0.129	f	0.023	±	0.007	bcd
T. michelianum		0.060	±	0.033	bcd	4.582	±	1.073	e	0.636	±	0.196	f	0.025	±	0.011	bcd
T. squarrosum		0.032	±	0.012	d	3.740	±	0.756	e	1.300	±	0.383	e	0.027	±	0.012	bcd
T. suaveolens		0.045	±	0.023	cd	8.462	±	3.262	d	1.019	±	0.254	e	0.022	±	0.011	cd
T. vesiculosum		0.032	±	0.005	d	5.138	±	1.125	e	0.588	±	0.217	f	0.013	±	0.002	d
Triticosecale		0.090	±	0.059	b	21.380	±	1.496	b	4.800	±	0.697	b	0.119	±	0.036	a
V. villosa		0.337	±	0.206	a	4.800	±	2.144	e	45.810	±	10.170	a	0.138	±	0.056	a
A. strigosa	7	0.078	±	0.009	bcd	26.970	±	2.519	a	5.464	±	0.508	b	0.136	±	0.020	a
L. multiflorum 2n		0.035	±	0.012	e	17.140	±	2.767	b	2.550	±	0.823	de	0.053	±	0.040	cd
L. multiflorum 4n		0.102	±	0.089	bcd	20.380	±	4.883	b	3.282	±	1.131	cd	0.079	±	0.025	bc
T. incarnatum		0.058	±	0.022	cde	3.791	±	0.524	d	0.436	±	0.220	h	0.031	±	0.010	de
T. michelianum		0.101	±	0.047	bc	4.809	±	1.252	d	0.682	±	0.199	gh	0.040	±	0.019	de
T. squarrosum		0.044	±	0.014	de	4.360	±	0.723	d	1.570	±	0.362	ef	0.032	±	0.009	de
T. suaveolens		0.110	±	0.047	b	10.940	±	3.675	c	1.395	±	0.552	f	0.044	±	0.016	de
T. vesiculosum		0.060	±	0.027	bcde	5.112	±	1.575	d	0.450	±	0.177	gh	0.024	±	0.011	e
Triticosecale		0.050	±	0.059	de	19.820	±	2.739	b	4.664	±	1.075	bc	0.089	±	0.039	b
V. villosa		0.440	±	0.284	a	4.291	±	1.972	d	48.880	±	10.940	a	0.148	±	0.057	a
A. strigosa	8	0.097	±	0.032	de	27.760	±	2.112	a	5.473	±	0.675	b	0.158	±	0.025	b
L. multiflorum 2n		0.048	±	0.025	e	17.900	±	2.997	c	2.562	±	0.994	c	0.091	±	0.060	cde

L. multiflorum 4n		0.082	±	0.020	de	22.850	±	4.393	b	4.000	±	1.275	b	0.097	±	0.023	bcd
T. incarnatum		0.083	±	0.062	de	3.567	±	0.952	e	0.692	±	0.227	f	0.048	±	0.018	e
T. michelianum		0.280	±	0.118	b	6.445	±	1.777	de	1.200	±	0.506	e	0.112	±	0.050	bcd
T. squarrosum		0.079	±	0.032	de	5.600	±	0.856	e	1.810	±	0.441	cd	0.061	±	0.028	de
T. suaveolens		0.233	±	0.185	bc	9.357	±	3.397	d	1.757	±	0.713	d	0.099	±	0.068	cde
T. vesiculosum		0.157	±	0.094	bcd	6.225	±	1.150	de	0.988	±	0.242	ef	0.058	±	0.042	de
Triticosecale		0.114	±	0.078	cd	22.230	±	2.225	b	5.709	±	1.189	b	0.143	±	0.049	bc
V. villosa		0.911	±	0.302	a	5.174	±	1.790	e	64.440	±	8.118	a	0.282	±	0.080	a
A. strigosa	9	0.066	±	0.011	fg	28.330	±	2.136	a	5.758	±	0.640	bc	0.164	±	0.029	bc
L. multiflorum 2n		0.041	±	0.016	g	19.590	±	3.040	c	2.633	±	0.604	d	0.083	±	0.019	ef
L. multiflorum 4n		0.054	±	0.016	fg	24.250	±	2.547	b	3.982	±	0.802	c	0.105	±	0.029	def
T. incarnatum		0.141	±	0.079	de	4.608	±	1.056	fg	0.983	±	0.490	e	0.083	±	0.033	f
T. michelianum		0.481	±	0.147	b	8.745	±	2.278	e	1.236	±	0.676	e	0.216	±	0.056	b
T. squarrosum		0.166	±	0.043	cd	7.530	±	0.987	ef	2.470	±	0.216	d	0.133	±	0.031	cde
T. suaveolens		0.287	±	0.160	c	11.760	±	3.732	d	2.352	±	0.969	d	0.124	±	0.051	cdef
T. vesiculosum		0.327	±	0.156	bc	7.862	±	1.501	ef	1.337	±	0.450	e	0.140	±	0.081	cdef
Triticosecale		0.082	±	0.018	ef	22.170	±	2.748	bc	6.108	±	0.891	b	0.152	±	0.025	bcd
V. villosa		1.051	±	0.353	a	3.984	±	1.480	g	72.470	±	8.198	a	0.316	±	0.080	a
T. incarnatum	10	0.384	±	0.200	c	4.992	±	0.931	c	1.058	±	0.193	d	0.179	±	0.063	b
T. michelianum		0.998	±	0.237	a	9.400	±	2.062	b	1.918	±	0.382	c	0.377	±	0.066	a
T. squarrosum		0.285	±	0.108	c	8.860	±	1.287	b	2.930	±	0.291	b	0.228	±	0.072	ab
T. suaveolens		0.433	±	0.166	bc	16.230	±	4.544	a	2.340	±	0.486	bc	0.260	±	0.068	ab
T. vesiculosum		0.500	±	0.236	bc	8.562	±	0.915	b	1.413	±	0.473	d	0.275	±	0.068	ab
V. villosa		0.767	±	0.214	ab	5.275	±	1.955	c	67.990	±	6.830	a	0.346	±	0.062	a

(Table S2 continuation)

Species	WAG	R/S				B				N				PGH			
A. strigosa		2.359	±	3.164	a												
L. multiflorum 2n		0.5015	±	0.1229	b												
L. multiflorum 4n	3	0.7966	±	0.2527	b												
T. suaveolens		4.376	±	12.70	b	0.000	±	0.000	b	5.091	±	5.449	b				
Triticosecale		0.9647	±	0.4605	ab												
V. villosa		0.6132	±	0.7071	b	0.818	±	0.751	a	27.820	±	7.068	a				
A. strigosa		1.664	±	0.7024	a												
L. multiflorum 2n		3.163	±	6.768	ab												
L. multiflorum 4n		1.078	±	0.3654	abc												
T. incarnatum		0.4479	±	0.07334	d	0.000	±	0.000	b	19.180	±	5.811	ab	1.636	±	0.505	cd
T. michelianum	4	0.4781	±	0.1028	d	0.000	±	0.000	b	7.600	±	1.838	bc	1.900	±	1.101	bc
T. squarrosum		0.6713	±	0.2032	bcd	0.000	±	0.000	b	7.600	±	4.624	bc	1.000	±	0.000	d
T. suaveolens		0.6607	±	0.1933	cd	0.000	±	0.000	b	9.579	±	9.094	c	1.222	±	0.441	d
T. vesiculosum		0.2435	±	0.1749	e	0.000	±	0.000	b	5.000	±	4.690	c	1.000	±	0.000	d
Triticosecale		1.355	±	0.1756	a												
V. villosa		0.4694	±	0.1166	d	1.045	±	1.174	a	31.410	±	15.100	a	5.000	±	0.000	a
A. strigosa		1.659	±	0.4031	a												
L. multiflorum 2n		1.856	±	2.608	a												
L. multiflorum 4n		1.759	±	2.238	a									1.545	±	0.688	cd
T. incarnatum		0.4110	±	0.05919	c	0.000	±	0.000	b	21.730	±	5.368	b	2.500	±	0.707	b
T. michelianum	5	0.3951	±	0.07529	c	0.000	±	0.000	b	14.600	±	4.671	bc	1.000	±	0.000	d
T. squarrosum		0.6446	±	0.1117	b	0.000	±	0.000	b	15.200	±	5.007	bc	2.125	±	0.991	bc
T. suaveolens		0.5559	±	0.1683	bc	0.000	±	0.000	b	23.050	±	14.640	b	2.000	±	0.577	bc
T. vesiculosum		0.4631	±	0.07690	bc	0.000	±	0.000	b	10.000	±	3.958	c				
Triticosecale		2.069	±	1.422	a												

V. villosa		0.4320	±	0.1362	c	2.476	±	1.632	a	49.000	±	18.010	a	5.000	±	0.000	a
A. strigosa		1.853	±	0.3865	a												
L. multiflorum 2n		1.075	±	0.6523	c												
L. multiflorum 4n		1.065	±	0.1219	bc												
T. incarnatum		0.4786	±	0.05671	d	0.000	±	0.000	d	27.360	±	14.560	bcde	1.864	±	0.839	d
T. michelianum	6	0.4303	±	0.06075	d	1.273	±	0.905	b	25.270	±	11.850	bcde	2.727	±	0.876	bcd
T. squarrosum		0.8160	±	0.1689	c	0.000	±	0.000	cd	20.700	±	6.360	bde	2.000	±	0.816	cd
T. suaveolens		0.4874	±	0.09117	d	0.000	±	0.000	d	34.620	±	18.800	bcd	2.750	±	1.034	bcd
T. vesiculosum		0.3965	±	0.04269	d	0.000	±	0.000	bcd	12.120	±	3.314	e	3.125	±	0.835	bc
Triticosecale		1.468	±	0.2774	ab												
V. villosa		0.4719	±	0.1395	d	3.682	±	1.585	a	113.100	±	35.730	a	5.000	±	0.000	a
A. strigosa		1.745	±	0.2598	a												
L. multiflorum 2n		1.511	±	0.9113	ab												
L. multiflorum 4n		0.9387	±	0.3522	bc												
T. incarnatum		0.5770	±	0.2095	de	0.364	±	0.674	bc	35.550	±	14.860	bc	1.845	±	0.966	f
T. michelianum	7	0.3881	±	0.04639	e	1.818	±	0.982	b	44.090	±	18.440	bc	3.091	±	0.701	bcde
T. squarrosum		0.7488	±	0.08994	cd	0.000	±	0.000	c	32.200	±	9.004	bc	2.000	±	0.816	ef
T. suaveolens		0.4095	±	0.08410	e	0.143	±	0.359	c	62.380	±	33.760	b	2.250	±	0.635	bdef
T. vesiculosum		0.3910	±	0.05788	e	0.250	±	0.463	bc	20.380	±	10.040	c	3.312	±	1.223	b
Triticosecale		1.822	±	0.6377	a												
V. villosa		0.4214	±	0.1894	e	5.136	±	1.935	a	118.300	±	53.160	a	5.000	±	0.000	a
A. strigosa		1.861	±	0.9723	a												
L. multiflorum 2n		2.257	±	2.002	a												
L. multiflorum 4n		1.200	±	0.1497	a												
T. incarnatum		0.6634	±	0.1647	b	0.500	±	1.168	d	27.000	±	10.770	c	1.450	±	0.643	ef
T. michelianum	8	0.3927	±	0.05251	cd	3.818	±	0.982	b	98.300	±	38.880	b	4.000	±	0.922	b
T. squarrosum		0.7612	±	0.06436	b	0.000	±	0.000	d	33.890	±	13.000	c	2.167	±	0.750	de
T. suaveolens		0.4625	±	0.1642	c	0.571	±	0.926	d	108.000	±	73.550	b	2.900	±	0.876	cd
T. vesiculosum		0.3567	±	0.04859	cd	1.125	±	1.126	cd	31.710	±	21.910	c	3.438	±	0.776	bc

Triticosecale		1.409	±	0.2614	a												
V. villosa		0.3274	±	0.07488	d	7.421	±	2.317	a	193.300	±	76.450	a	5.000	±	0.000	a
A. strigosa	9	2.495	±	0.3365	a												
L. multiflorum 2n		2.146	±	0.5027	a												
L. multiflorum 4n		2.002	±	0.3447	a												
T. incarnatum		0.6595	±	0.2083	bc	0.667	±	0.778	d	40.080	±	15.480	b	2.625	±	1.384	cd
T. michelianum		0.4592	±	0.05601	cd	4.091	±	1.514	b	100.500	±	29.190	ab	3.318	±	0.513	bc
T. squarrosum		0.8089	±	0.05902	b	0.400	±	0.699	d	42.700	±	9.031	b	2.900	±	0.394	bcd
T. suaveolens		0.4773	±	0.1074	d	0.435	±	0.896	d	160.500	±	128.600	a	2.150	±	0.669	d
T. vesiculosum		0.4123	±	0.07805	de	2.000	±	1.069	cd	45.000	±	21.710	b	4.000	±	0.926	ab
Triticosecale		1.891	±	0.3066	a												
V. villosa		0.3509	±	0.1575	e	7.591	±	1.843	a	210.900	±	115.700	a	5.000	±	0.000	a
T. incarnatum	10	0.6272	±	0.2218	b	1.833	±	1.267	bc	46.500	±	14.800	b	4.542	±	0.582	a
T. michelianum		0.4060	±	0.07515	c	7.000	±	0.775	a	119.500	±	32.970	a	3.727	±	0.754	bc
T. squarrosum		0.8224	±	0.1076	a	1.100	±	0.876	bc	48.200	±	14.700	b	3.150	±	0.337	cd
T. suaveolens		0.6178	±	0.1174	ab	0.300	±	0.675	c	64.500	±	24.770	b	2.350	±	0.914	d
T. vesiculosum		0.4945	±	0.05479	bc	2.500	±	1.512	b	54.500	±	15.960	b	4.500	±	0.707	ab
V. villosa		0.4957	±	0.1688	bc	6.583	±	2.109	a	159.200	±	57.160	a	5.000	±	0.000	a

Table S3: Average morphological and standard deviations values for 10 annual forage species, measured 9 or 10 weeks after germination (WAG). Letters in common indicate that the values are not statistically significant (P<0.05, Tukey test). Traits' abbreviations: DRL – dry root length (cm), LAR - leaf area (mm^2), LDMC - leaf dry matter content (mg g^{-1}RDW - root fraction dry weight (g), RL - root length (cm), SLA - specific leaf area (mm^2 mg^{-1}), SRL - secondary root length (cm), SRRL - secondary root length to root length ratio

Species	WAG	DRL				LAR				LDMC				RL			
A. strigosa	9	0.003	±	0.001	bc	1021.000	±	142.600	b	536.000	±	75.400	a	66.960	±	21.070	ab
L. multiflorum 2n		0.002	±	0.001	c	437.000	±	128.600	e	188.900	±	31.620	b	47.960	±	12.820	bcde
L. multiflorum 4n		0.002	±	0.001	c	698.200	±	140.500	cd	179.900	±	22.940	b	57.450	±	12.830	abcd
T. suaveolens		0.004	±	0.001	ab	2168.000	±	511.300	a	130.200	±	8.775	d	40.720	±	14.340	ce

Triticosecale		0.002	±	0.001	c	569.300	±	94.790	de	197.100	±	16.930	b	79.800	±	19.490	a
V. villosa		0.005	±	0.002	a	997.900	±	392.400	b	151.300	±	32.590	cd	71.030	±	23.180	a
T. incarnatum		0.003	±	0.001	b	847.500	±	181.200	bc	177.000	±	16.150	a	60.500	±	14.540	a
T. michelianum		0.006	±	0.002	a	679.600	±	163.000	c	137.400	±	19.780	b	62.030	±	21.030	a
T. squarrosum		0.006	±	0.002	a	1070.000	±	185.100	b	183.400	±	27.170	a	40.110	±	13.960	b
T. suaveolens	10	0.005	±	0.002	ab	2489.000	±	425.100	a	118.900	±	23.510	b	57.500	±	19.390	ab
T. vesiculosum		0.005	±	0.002	ab	1005.000	±	164.600	b	146.700	±	20.270	ab	48.700	±	10.090	ab
V. villosa		0.006	±	0.002	a	419.800	±	96.180	d	149.400	±	29.480	b	55.840	±	13.040	a

(Table S3 continuation)

Species	WAG	SLA				SRL				SRRL			
A. strigosa		9.745	±	11.670	e	21.870	±	3.451	a	0.353	±	0.096	abc
L. multiflorum 2n		29.000	±	7.518	cd	19.760	±	2.954	ab	0.437	±	0.123	a
L. multiflorum 4n		26.250	±	5.005	d					0.381	±	0.133	ab
T. suaveolens	9	43.150	±	4.181	b					0.450	±	0.109	a
Triticosecale		29.220	±	3.277	cd	17.950	±	3.207	b	0.205	±	0.067	c
V. villosa		51.230	±	9.811	a					0.292	±	0.120	bc
T. incarnatum		38.570	±	5.641	b	15.740	±	1.712	c	0.271	±	0.053	c
T. michelianum		45.420	±	11.510	ab	20.710	±	2.151	ab	0.372	±	0.134	bc
T. squarrosum		42.770	±	8.101	ab	19.530	±	1.511	ab	0.524	±	0.131	a
T. suaveolens	10	53.260	±	18.630	a	22.320	±	2.543	a	0.424	±	0.133	ab
T. vesiculosum		45.160	±	8.149	ab	18.150	±	3.642	bc	0.375	±	0.050	abc
V. villosa		55.410	±	11.180	a	21.500	±	3.354	a	0.401	±	0.126	abc

Table S4: Analysis of variance for the physiological traits measured on 10 annual forage species under two water regimens (well-watered and water deficit). Trait's abbreviations: A_{400}: net photosynthesis or net CO_2 assimilation rate (µmol CO_2 m^{-2} s^{-1}) of plants grown at 400 µmol m^{-2} s^{-1} of photosynthetically active radiation (PAR); A_{782}: net photosynthesis or net CO_2 assimilation rate (µmol CO_2 m^{-2} s^{-1}) of plant grown at 782 µmol m^{-2} s^{-1} PAR; Cha/Chb: chlorophyll *a* by the chlorophyll *b* ratio; Cha+Chb: total chlorophyll content (mg g^{-1}); Cha+Chb/Ccx: total chlorophyll content by carotenoids ratio; Cha: chlorophyll *a* (mg g^{-1}); Chb: chlorophyll *b* (mg g^{-1}); Ccx: carotenoids (mg g^{-1}); E_{400}: transpiration rate (mmol H_2O m^{-2} s^{-1}) of plant grown at 400 µmol m^{-2} s^{-1} PAR; E_{782}: transpiration rate (mmol H_2O m^{-2} s^{-1}) of plants grown at 782 µmol m^{-2} s^{-1} PAR; Fv/Fm: maximum quantum yield of photochemistry in Photosystem II; Fv/Fo: maximum quantum yield of photochemistry in photosystem II normalized by the minimum fluorescence; gs_{400}: stomatal conductance (mol CO_2 m^{-2} s^{-1}) of plants grown at 400 µmol m^{-2} s^{-1} PAR; gs_{782}: stomatal conductance (mol CO_2 m^{-2} s^{-1}) of plants grown at 782 µmol m^{-2} s^{-1} PAR; PI_{ABS}: performance index; RWC: leaf relative water content (%).

Trait	Source of variation	d.f.	s.s.	m.s.	v.r.	F pr.
A_{400} (Box-Cox transformation of A_400*+0.5* with parameter 0.25)	Species	9	422.7987	46.9776	71.52	<.001
	Treatment	1	43.448	43.448	66.15	<.001
	Species.Treatment	9	14.4892	1.6099	2.45	0.01
	Residual	348	228.5717	0.6568		
	Total	367	647.6987			
A_{400}/E (Box-Cox transformation of A_400/E with parameter 0.5)	Species	9	195.3648	21.7072	58.75	<.001
	Treat	1	4.9135	4.9135	13.30	<.001
	Species.Treatment	9	15.6076	1.7342	4.69	<.001
	Residual	347	128.2066	0.3695		
	Total	366	305.5127			
A_{400}/gs (Box-Cox transformation of A_400/gs with parameter 0.5)	Species	9	4390.316	487.813	59.22	<.001
	Treat	1	263.223	263.223	31.95	<.001
	Species.Treatment	9	118.114	13.124	1.59	0.116
	Residual	334	2751.401	8.238		
	Total	353	6686.933			
A_{782} (Box-Cox transformation of A_782*+0.5* with parameter 0.25)	Species	9	502.2412	55.8046	63.5	<.001
	Treatment	1	42.266	42.266	48.1	<.001
	Species.Treatment	9	20.9532	2.3281	2.65	0.006
	Residual	348	305.8157	0.8788		
	Total	367	800.4222			

A_{782}/E (Box-Cox transformation of A/782_E with parameter 0.5)	Species	9	350.5930	38.9548	102.08	<.001
	Treat	1	5.1467	5.1467	13.49	<.001
	Species.Treat	9	12.1857	1.3540	3.55	<.001
	Residual	345	131.6557	0.3816		
	Total	364	444.6111			
A_{782}/gs (Box-Cox transformation of A_782/gs with parameter 0.5)	Species	9	9402.05	1044.67	68.40	<.001
	Treat	1	453.50	453.50	29.69	<.001
	Species.Treat	9	320.86	35.65	2.33	0.015
	Residual	338	5162.12	15.27		
	Total	357	13714.65			
Cha+Chb (Box-Cox transformation of Cha+Chb with parameter 0.5)	Species	9	1066.671	118.519	33.41	<.001
	Treatment	1	2.766	2.766	0.78	0.378
	Species.Treatment	9	33.608	3.734	1.05	0.398
	Residual	351	1245.1	3.547		
	Total	370	2235.056			
Cha+Chb/Ccx (Box-Cox transformation of ChaChb/Ccx with parameter 0.25)	Species	9	9.1053	1.0117	11.66	<.001
	Treatment	1	0.06807	0.06807	0.78	0.376
	Species.Treatment	9	1.51844	0.16872	1.94	0.045
	Residual	374	32.44552	0.08675		
	Total	393	42.25573			
Cha (Box-Cox transformation of Cha with parameter 0.5)	Species	9	504.545	56.061	32.28	<.001
	Treatment	1	0.447	0.447	0.26	0.612
	Species.Treatment	9	14.166	1.574	0.91	0.52
	Residual	351	609.624	1.737		
	Total	370	1074.665			
Cha/Chb (Box-Cox transformation of Cha/Chb with parameter 2.25)	Species	9	154.351	17.15	12.39	<.001
	Treatment	1	2.751	2.751	1.99	0.160
	Species.Treatment	9	14.077	1.564	1.13	0.341
	Residual	374	517.869	1.385		
	Total	393	677.618			
Chb (Box-Cox	Species	9	137.3934	15.2659	24.81	<.001

transformation of Chb with parameter 0.5)	Treatment	1	0.6021	0.6021	0.98	0.323
	Species.Treatment	9	4.0181	0.4465	0.73	0.685
	Residual	351	215.9862	0.6153		
	Total	370	342.7517			
Ccx (Box-Cox transformation of Ccx with parameter 0.5)	Species	9	94.5377	10.5042	30.15	<.001
	Treatment	1	0.0165	0.0165	0.05	0.828
	Species.Treatment	9	2.8331	0.3148	0.9	0.522
	Residual	348	121.2435	0.3484		
	Total	367	207.6554			
E_{400} (Box-Cox transformation of E_400 with parameter 0.5)	Species	9	196.0907	21.7879	45.85	<.001
	Treatment	1	65.0834	65.0834	136.97	<.001
	Species.Treatment	9	29.5702	3.2856	6.91	<.001
	Residual	347	164.8779	0.4752		
	Total	366	417.7656			
E_{782} (Box-Cox transformation of E_782 with parameter 0.5)	Species	9	164.5027	18.2781	33.48	<.001
	Treatment	1	60.833	60.833	111.44	<.001
	Species.Treatment	9	33.8397	3.76	6.89	<.001
	Residual	345	188.322	0.5459		
	Total	364	413.9491			
Fv/Fm (Box-Cox transformation of Fv/Fm with parameter 4)	Species	9	0.109006	0.012112	82.99	<.001
	Treatment	1	9E-07	9E-07	0.01	0.938
	Species.Treatment	9	0.001152	0.000128	0.88	0.546
	Residual	337	0.049184	0.000146		
	Total	356	0.139681			
Fv/Fo (Box-Cox transformation of Fv/Fo with parameter 2)	Species	9	2384.921	264.991	78.97	<.001
	Treatment	1	2.005	2.005	0.6	0.44
	Species.Treatment	9	16.077	1.786	0.53	0.851
	Residual	345	1157.748	3.356		
	Total	364	3192.928			
PI_{ABS} (Box-Cox transformation	Species	9	132.2769	14.6974	31.32	<.001
	Treatment	1	0.1717	0.1717	0.37	0.546

of PI_{ABS} with parameter 0.5)	Species.Treatment	9	3.1688	0.3521	0.75	0.663
	Residual	343	160.9826	0.4693		
	Total	362	274.819			
RWC (Box-Cox transformation of RWC with parameter 3.25)	Species	9	4.28E+12	4.75E+11	23.14	<.001
	Treatment	1	1.24E+09	1.24E+09	0.06	0.806
	Species.Treatment	9	1.41E+11	1.56E+10	0.76	0.652
	Residual	377	7.74E+12	2.05E+10		
	Total	396	1.17E+13			
gs_{400} (Box-Cox transformation of gs_400_0_5 with parameter -3)	Species	9	82.0859	9.1207	58.41	<.001
	Treatment	1	29.0103	29.0103	185.8	<.001
	Species.Treatment	9	12.6079	1.4009	8.97	<.001
	Residual	345	53.8669	0.1561		
	Total	364	163.0167			
gs_{782} (Box-Cox transformation of gs_782_0_5 with parameter -2.5)	Species	9	43.5436	4.8382	42.46	<.001
	Treatment	1	15.2473	15.2473	133.82	<.001
	Species.Treatment	9	8.11	0.9011	7.91	<.001
	Residual	344	39.1945	0.1139		
	Total	363	98.1485			

Table S5: Average and standard deviation values for physiological traits measured under well-watered (WW) and water deficit (WD) conditions in 10 annual forage species. The same letters along the columns indicate that the differences among values are not statistically significant (P<0.05, Tukey test, performed on transformed data). Trait's abbreviations: A_{400}: net photosynthesis or net CO_2 assimilation rate (µmol CO_2 m^{-2} s^{-1}) of plants grown at 400 µmol m^{-2} s^{-1} of photosynthetically active radiation (PAR); A_{782}: net photosynthesis or net CO_2 assimilation rate (µmol CO_2 m^{-2} s^{-1}) of plant grown at 782 µmol m^{-2} s^{-1} PAR; Cha/Chb: chlorophyll *a* by the chlorophyll *b* ratio; Cha+Chb: total chlorophyll content (mg g^{-1}); Cha+Chb/Ccx: total chlorophyll content by carotenoids ratio; Cha: chlorophyll *a* (mg g^{-1}); Chb: chlorophyll *b* (mg g^{-1}); Ccx: carotenoids (mg g^{-1}); E_{400}: transpiration rate (mmol H_2O m^{-2} s^{-1}) of plant grown at 400 µmol m^{-2} s^{-1} PAR; E_{782}: transpiration rate (mmol H_2O m^{-2} s^{-1}) of plants grown at 782 µmol m^{-2} s^{-1} PAR; Fv/Fm: maximum quantum yield of photochemistry in photosystem II; Fv/Fo: maximum quantum yield of photochemistry in photosystem II normalized by the minimum fluorescence; gs_{400}: stomatal conductance (mol CO_2 m^{-2} s^{-1}) of plants grown at 400 µmol m^{-2} s^{-1} PAR; gs_{782}: stomatal conductance (mol CO_2 m^{-2} s^{-1}) of plants grown at 782 µmol m^{-2} s^{-1} PAR; PI_{ABS}: performance index; RWC: leaf relative water content (%).

Species	Treatment	RWC				A_{400}				E_{400}				gs_{400}				A_{782}			
A. strigosa		93.142	±	3.017	a	0.788	±	0.693	b	1.164	±	0.547	bcd	0.036	±	0.026	cde	2.177	±	1.538	bc
L. multiflorum 2n		87.525	±	7.597	ab	0.693	±	0.860	b	2.476	±	0.978	a	0.081	±	0.037	abcd	1.319	±	1.299	d
L. multiflorum 4n		88.061	±	9.817	a	0.684	±	0.926	b	3.098	±	1.207	a	0.101	±	0.052	a	1.963	±	2.615	d
T. incarnatum		79.169	±	4.184	c	4.856	±	2.307	a	1.931	±	0.981	abcd	0.101	±	0.071	ab	7.935	±	2.643	ab
T. michelianum	WD	82.132	±	3.883	bc	3.796	±	1.756	a	2.005	±	0.724	abc	0.084	±	0.037	abcd	6.497	±	2.864	ab
T. squarrosum		81.164	±	4.923	bc	5.188	±	2.532	a	2.365	±	1.101	a	0.111	±	0.065	a	9.446	±	4.884	a
T. suaveolens		81.795	±	6.173	bc	4.870	±	3.720	a	2.357	±	1.749	a	0.110	±	0.095	abc	8.886	±	5.884	a
T. vesiculosum		80.308	±	5.782	c	4.947	±	3.069	a	2.171	±	1.206	ab	0.097	±	0.071	abc	8.870	±	5.001	a
Triticosecale		92.087	±	5.020	a	0.391	±	0.520	b	0.845	±	0.350	cd	0.017	±	0.019	cde	2.215	±	1.645	bc
V. villosa		78.201	±	11.574	c	0.176	±	0.279	b	0.443	±	0.317	d	0.008	±	0.012	e	1.040	±	1.398	d
A. strigosa		90.906	±	10.665	a	0.837	±	0.811	b	1.079	±	0.524	d	0.033	±	0.030	d	1.714	±	1.394	b
L. multiflorum 2n		83.745	±	12.101	abc	1.077	±	1.284	b	3.161	±	1.050	bc	0.110	±	0.048	c	2.702	±	3.805	b
L. multiflorum 4n		86.954	±	7.787	ab	1.745	±	1.311	b	3.693	±	1.042	bc	0.143	±	0.092	bc	3.004	±	2.164	b
T. incarnatum		76.296	±	3.443	c	7.348	±	3.680	a	4.418	±	1.403	ab	0.291	±	0.149	a	12.345	±	5.325	a
T. michelianum	WW	82.346	±	3.901	bc	8.250	±	3.541	a	5.461	±	1.795	a	0.280	±	0.091	a	14.400	±	5.396	a
T. squarrosum		82.287	±	7.002	bc	5.452	±	1.699	a	2.564	±	0.833	c	0.126	±	0.053	bc	9.559	±	2.855	a
T. suaveolens		83.574	±	7.480	bc	7.550	±	2.428	a	3.911	±	1.087	b	0.310	±	0.156	a	12.641	±	3.617	a
T. vesiculosum		80.974	±	5.358	bc	6.792	±	4.020	a	3.753	±	1.403	bc	0.213	±	0.110	ab	11.269	±	6.316	a
Triticosecale		91.469	±	4.106	a	1.096	±	1.017	b	1.159	±	0.423	d	0.030	±	0.022	d	2.939	±	1.808	b

V. villosa	77.431	±	12.586	c	1.953	±	1.186	b	1.194	±	0.646	d	0.046	±	0.030	d	3.346	±	1.642	b

(Table S5 continuation)

Species	Treatment	E_{782}				gs_{782}				Fv/Fm			
A. strigosa	WD	1.973	±	0.774	b	0.070	±	0.037	bcde	0.726	±	0.061	de
L. multiflorum 2n		2.877	±	1.379	ab	0.096	±	0.057	abcde	0.719	±	0.036	e
L. multiflorum 4n		3.478	±	1.398	a	0.117	±	0.062	abcd	0.715	±	0.039	e
T. incarnatum		2.500	±	0.888	ab	0.119	±	0.068	abc	0.812	±	0.014	a
T. michelianum		2.474	±	1.011	ab	0.106	±	0.059	abcde	0.801	±	0.015	ab
T. squarrosum		3.135	±	1.503	ab	0.158	±	0.095	a	0.814	±	0.014	a
T. suaveolens		2.845	±	1.667	ab	0.150	±	0.113	a	0.801	±	0.017	a
T. vesiculosum		2.788	±	1.419	ab	0.130	±	0.085	ab	0.799	±	0.028	ab
Triticosecale		1.685	±	0.749	b	0.050	±	0.032	cdef	0.761	±	0.022	cd
V. villosa		0.617	±	0.607	c	0.017	±	0.024	f	0.778	±	0.024	bc
A. strigosa	WW	1.589	±	0.791	e	0.054	±	0.044	e	0.735	±	0.054	cd
L. multiflorum 2n		3.883	±	1.341	bc	0.142	±	0.095	cd	0.692	±	0.045	e
L. multiflorum 4n		3.861	±	0.985	bc	0.128	±	0.052	cd	0.718	±	0.046	de
T. incarnatum		5.163	±	1.662	ab	0.356	±	0.191	a	0.812	±	0.014	a
T. michelianum		6.359	±	2.077	a	0.321	±	0.107	a	0.808	±	0.013	a
T. squarrosum		3.257	±	1.023	cd	0.165	±	0.070	bc	0.813	±	0.011	a
T. suaveolens		4.524	±	1.226	b	0.366	±	0.174	a	0.798	±	0.023	a
T. vesiculosum		4.469	±	1.641	bc	0.263	±	0.134	ab	0.798	±	0.036	a
Triticosecale		2.139	±	0.676	de	0.075	±	0.030	de	0.760	±	0.032	bc
V. villosa		1.484	±	0.771	e	0.057	±	0.036	e	0.787	±	0.024	ab

(Table S5 continuation)

Species	Treatment	Fv/Fo				PI$_{ABS}$				Cha				Chb				Ccx			
A. strigosa	WD	2.290	±	1.016	g	1.635	±	0.900	d	4.936	±	4.371	b	2.148	±	1.970	c	1.238	±	0.935	e
L. multiflorum 2n		2.622	±	0.482	fg	3.314	±	1.168	bc	5.507	±	2.762	b	2.272	±	1.099	bc	1.346	±	0.711	cde
L. multiflorum 4n		2.573	±	0.469	fg	2.474	±	1.019	cd	5.344	±	3.911	b	2.020	±	1.472	c	1.353	±	0.963	de
T. incarnatum		4.346	±	0.405	a	5.107	±	0.726	a	7.649	±	0.869	ab	2.756	±	0.477	abc	1.688	±	0.299	bcde
T. michelianum		4.047	±	0.371	abcd	4.728	±	0.733	a	10.641	±	4.018	a	3.821	±	1.552	ab	2.154	±	0.718	abcd
T. squarrosum		4.406	±	0.381	a	4.989	±	1.354	a	10.918	±	3.826	a	3.854	±	1.350	a	2.261	±	0.754	ab
T. suaveolens		4.062	±	0.423	abc	3.576	±	1.221	b	10.682	±	4.000	a	3.855	±	1.386	a	2.475	±	0.968	ab
T. vesiculosum		4.061	±	0.580	ab	4.048	±	1.341	ab	10.575	±	2.965	a	3.669	±	1.050	ab	2.224	±	0.704	abc
Triticosecale		3.227	±	0.395	ef	3.298	±	1.053	bc	5.517	±	2.882	b	1.943	±	0.941	c	1.271	±	0.723	e
V. villosa		3.566	±	0.490	bcde	4.055	±	1.355	ab	13.135	±	6.635	a	4.679	±	2.024	a	3.052	±	0.843	a
A. strigosa	WW	2.563	±	0.906	de	1.476	±	0.617	d	4.726	±	3.142	cd	1.800	±	1.086	cd	1.272	±	0.718	de
L. multiflorum 2n		2.328	±	0.533	e	2.886	±	1.255	c	4.028	±	2.357	d	1.747	±	0.941	cd	1.175	±	0.755	de
L. multiflorum 4n		2.646	±	0.592	de	2.997	±	1.977	c	3.283	±	1.761	d	1.623	±	1.009	d	0.788	±	0.371	e
T. incarnatum		4.347	±	0.383	ab	4.987	±	2.005	ab	8.184	±	1.952	abc	2.858	±	0.726	abc	1.738	±	0.512	bcd
T. michelianum		4.235	±	0.333	ab	5.242	±	1.677	a	10.888	±	8.669	ab	2.950	±	0.905	abc	2.093	±	0.625	abc
T. squarrosum		4.393	±	0.339	a	4.840	±	1.227	ab	11.598	±	2.812	a	4.007	±	0.953	a	2.265	±	0.640	abc
T. suaveolens		4.033	±	0.562	ab	3.717	±	1.712	bc	11.287	±	4.798	a	4.011	±	1.770	a	2.555	±	1.056	ab
T. vesiculosum		4.087	±	0.709	ab	4.183	±	1.643	abc	10.696	±	4.015	ab	3.642	±	1.407	ab	2.418	±	0.859	ab
Triticosecale		3.236	±	0.528	cd	2.875	±	0.926	c	6.481	±	3.289	bcd	2.303	±	1.198	bcd	1.490	±	0.786	cde
V. villosa		3.758	±	0.497	bc	3.381	±	1.054	abc	12.174	±	5.945	a	4.403	±	2.381	a	3.165	±	1.427	a

(Table S5 continuation)

Species	Treatment	Cha+Chb				Cha/Chb				Cha+Chb/Ccx			
A. strigosa	WD	11.159	±	9.902	b	2.345	±	0.401	cd	8.824	±	1.961	bc
L. multiflorum 2n		12.381	±	6.170	b	2.438	±	0.258	bcd	9.350	±	1.721	abc
L. multiflorum 4n		11.909	±	8.640	b	2.516	±	0.599	abcd	8.985	±	1.983	bc
T. incarnatum		16.968	±	1.992	ab	2.814	±	0.235	ab	10.326	±	1.368	ab
T. michelianum		23.597	±	8.922	a	2.840	±	0.362	a	10.954	±	1.970	a
T. squarrosum		24.173	±	8.450	a	2.840	±	0.229	a	10.726	±	1.538	a
T. suaveolens		23.698	±	8.821	a	2.770	±	0.242	abc	9.756	±	1.414	ab
T. vesiculosum		23.375	±	6.537	a	2.842	±	0.325	a	10.971	±	1.695	a
Triticosecale		12.212	±	6.305	b	2.848	±	0.457	a	9.729	±	1.238	ab
V. villosa		29.104	±	14.478	a	2.760	±	0.305	abc	8.067	±	0.911	c
A. strigosa	WW	10.540	±	6.931	cd	2.526	±	0.454	bc	8.207	±	1.860	d
L. multiflorum 2n		9.104	±	5.228	cd	2.360	±	0.598	c	8.629	±	2.773	cd
L. multiflorum 4n		7.534	±	4.066	d	2.228	±	0.578	c	9.439	±	1.985	bcd
T. incarnatum		18.102	±	4.321	ab	2.890	±	0.243	ab	10.620	±	1.643	ab
T. michelianum		19.431	±	5.858	ab	2.984	±	0.226	a	9.647	±	1.271	abcd
T. squarrosum		25.304	±	6.149	a	2.863	±	0.159	ab	11.324	±	1.534	a
T. suaveolens		25.005	±	10.647	a	2.841	±	0.232	ab	9.896	±	1.547	abc
T. vesiculosum		23.606	±	8.857	a	2.963	±	0.267	a	9.734	±	1.725	abcd
Triticosecale		14.358	±	7.291	bc	2.856	±	0.308	ab	9.706	±	1.132	abcd
V. villosa		27.014	±	13.291	a	2.819	±	0.358	ab	8.708	±	1.289	cd

(Table S5 continuation)

Species	Treatment	WUE=A_{400}/E				WUE=A_{782}/E				WUEi=A_{400}/gs				WUEi=A_{782}/gs			
A. strigosa	WD	0.701	±	0.715	cd	1.079	±	0.599	bc	26.400	±	21.201	b	33.498	±	19.541	b
L. multiflorum 2n		0.306	±	0.370	de	0.484	±	0.472	cd	9.724	±	11.532	b	15.889	±	15.881	b
L. multiflorum 4n		0.217	±	0.247	e	0.507	±	0.455	d	6.554	±	7.024	b	15.811	±	12.253	b
T. incarnatum		2.530	±	0.217	a	3.192	±	0.077	a	52.867	±	13.426	a	72.813	±	16.204	a
T. michelianum		1.910	±	0.619	ab	2.617	±	0.510	a	48.019	±	18.682	a	64.261	±	14.206	a
T. squarrosum		2.130	±	0.682	ab	2.866	±	0.817	a	48.345	±	16.740	a	60.647	±	19.353	a
T. suaveolens		1.992	±	0.881	ab	2.819	±	0.876	a	43.301	±	18.377	a	57.487	±	16.994	a
T. vesiculosum		2.306	±	0.705	ab	3.159	±	0.594	a	57.921	±	21.058	a	73.882	±	14.602	a
Triticosecale		0.441	±	0.471	de	1.239	±	0.670	b	21.361	±	18.351	b	45.977	±	27.289	b
V. villosa		0.343	±	0.402	bc	1.130	±	0.926	a	13.875	±	16.242	b	64.979	±	13.332	b
A. strigosa	WW	0.675	±	0.475	d	0.935	±	0.576	de	25.079	±	12.906	b	31.811	±	15.659	b
L. multiflorum 2n		0.354	±	0.441	e	0.616	±	0.604	e	11.223	±	16.319	b	17.322	±	13.174	b
L. multiflorum 4n		0.446	±	0.334	e	0.681	±	0.383	e	12.680	±	9.215	b	22.931	±	12.957	b
T. incarnatum		1.677	±	0.683	bc	2.418	±	0.730	abc	28.296	±	14.646	a	39.945	±	16.938	a
T. michelianum		1.490	±	0.509	cd	2.255	±	0.412	c	29.060	±	10.185	a	44.736	±	7.307	a
T. squarrosum		2.205	±	0.531	a	2.962	±	0.331	a	47.639	±	14.556	a	62.103	±	12.165	a
T. suaveolens		1.953	±	0.546	ab	2.801	±	0.584	ab	27.350	±	9.681	a	38.223	±	9.703	a
T. vesiculosum		1.736	±	0.791	abc	2.374	±	0.943	bc	31.427	±	15.299	a	41.742	±	17.353	a
Triticosecale		0.839	±	0.558	d	1.300	±	0.602	d	32.405	±	15.286	b	37.320	±	15.810	b
V. villosa		1.523	±	0.554	ab	2.276	±	0.463	abc	43.392	±	12.878	b	65.497	±	19.131	b
